# Effect of Low-Fat Diet in Obese Mice Lacking Toll-like Receptors

**DOI:** 10.3390/nu10101464

**Published:** 2018-10-09

**Authors:** Cheng-Shyuan Rau, Shao-Chun Wu, Tsu-Hsiang Lu, Yi-Chan Wu, Chia-Jung Wu, Peng-Chen Chien, Pao-Jen Kuo, Chia-Wei Lin, Chia-Wen Tsai, Ching-Hua Hsieh

**Affiliations:** 1 Department of Neurosurgery, Kaohsiung Chang Gung Memorial Hospital and Chang Gung University College of Medicine, No. 123, Ta-Pei Road, Niao-Song District, Kaohsiung City 833, Taiwan; ersh2127@adm.cgmh.org.tw; 2 Department of Anesthesiology, Kaohsiung Chang Gung Memorial Hospital and Chang Gung University College of Medicine, No. 123, Ta-Pei Road, Niao-Song District, Kaohsiung City 833, Taiwan; shaochunwu@gmail.com; 3 Department of Plastic Surgery, Kaohsiung Chang Gung Memorial Hospital and Chang Gung University College of Medicine, No. 123, Ta-Pei Road, Niao-Song District, Kaohsiung City 833, Taiwan; rabbit670326@yahoo.com.tw (T.-H.L.); janewu0922@gmail.com (Y.-C.W.); alice8818@yahoo.com.tw (C.-J.W.); VENU_CHIEN@hotmail.com (P.-C.C.); bow110470@gmail.com (P.-J.K.); sallylin1201@gmail.com (C.-W.L.); flying011401@gmail.com (C.-W.T.)

**Keywords:** diet-induced obesity, high-fat diet, low-fat diet, toll-like receptor, weight reduction

## Abstract

*Background:* This study aimed at assessing the effect of a low-fat diet (LFD) in obese mice lacking toll–like receptors (Tlr) and understanding the expression and regulation of microRNAs during weight reduction. *Methods:* C57BL/6, Tlr5^−/−^, Tlr2^−/−^ and Tlr4^−/−^ mice were used in this study. A group of mice were fed with a high-fat diet (HFD) (58% kcal) for 12 weeks to induce obesity (diet-induced obesity, DIO). Another group that had been fed with HFD for eight weeks (obese mice) were switched to a low-fat diet (LFD) (10.5% kcal) for the next four weeks to reduce their body weight. The control mice were fed with a standard AIN-76A diet for the entire 12 weeks. The body weight of the mice was measured weekly. At the end of the experiment, epididymal fat weight and adipocyte size were measured. The differentially expressed miRNAs in the fat tissue was determined by next-generation sequencing with real-time quantitative reverse transcription polymerase chain reaction (RT–qPCR). Target prediction and functional annotation of miRNAs were performed using miRSystem database. *Results:* Switching to LFD significantly reduced the body weight and epididymal fat mass in the HFD-fed C57BL/6 and Tlr5^−/−^ mice but not in Tlr2^−/−^ and Tlr4^−/−^ mice. Weight reduction significantly decreased the size of adipocytes in C57BL/6 but not in the *Tlr* knockout mice. In Tlr2^−/−^ and Tlr4^−/−^ mice, feeding with HFD and the subsequent weight reduction resulted in an aberrant miRNA expression in the epididymal fat tissue unlike in C57BL/6 and Tlr5^−/−^. However, target prediction and functional annotation by miRSystem database revealed that all the top 10 Kyoto Encyclopedia of Genes and Genomes (KEGG) database pathways of the dysregulated miRNAs during weight reduction in the C57BL/6 mice were also found in the regulated pathways of Tlr5^−/−^, Tlr2^−/−^, and Tlr4^−/−^ strains. However, among these pathways, gene sets involved in arginine and proline metabolism and glutathione metabolism were mainly involved in the *Tlr* knockout mice but not in the C57BL/6 mice. *Conclusions:* In this study, we demonstrated that feeding of LFD leads to significant body weight reduction in C57BL/6 and Tlr5^−/−^ mice, but not in Tlr2^−/−^ and Tlr4^−/−^ mice. Significant reduction in the size of adipocytes of epididymal fat was only found in C57BL/6, but not in Tlr5^−/−^, Tlr2^−/−^, and Tlr4^−/−^ mice. The dysregulated miRNAs in Tlr2^−/−^ and Tlr4^−/−^ mice were different from those in C57BL/6 and Tlr5^−/−^ strains. Among those miRNA-regulated pathways, arginine and proline metabolism as well as glutathione metabolism may have important roles in the *Tlr* knockout mice rather than in C57BL/6 mice.

## 1. Introduction

Obesity is now generally recognized as a disease associated with chronic inflammation response [1,2,3]. Uptake of a high-fat diet leads to the synthesis and release of adipokines and proinflammatory cytokines in the adipose tissue [1]. In addition, the increased adiposity propagates the synthesis of pro-inflammatory cytokines and presents a negative impact on the liver [4], muscle [5], and bone [5,6]. These associated inflammatory responses were demonstrated as important mechanisms mediating insulin resistance and hepatic steatosis [7,8]. There is convincing evidence that these pathways are related to the activation of toll-like receptors (Tlrs) [3,9,10], which are pattern-recognition receptors that detect microbial components to provide the first line of host defense against infections [11,12,13]. Upon stimulation, the Tlrs recruit the interleukin-1 receptor (IL1R1)-associated protein kinases that are linked with downstream nuclear factor –kappa B (NF-κB) activation and upregulated expression of cytokines and chemokines via MyD88-dependent and -independent cascades [11,12,13,14]. The extent of the obesity-induced upregulation of most Tlr genes and related proinflammatory signaling cascades is much greater in the epididymal adipose tissues than in the subcutaneous fat tissues of mice with diet-induced obesity (DIO) [14].

The obesity induced by a high-fat diet is associated with an increased expression of Tlr1–9 and Tlr11–13 in the adipose tissues [14]. Mice fed with a high-fat diet (HFD) have an elevated level of free fatty acids (FFAs), which further activates the expression of Tlr2 [15,16,17], Tlr4 [15,17,18,19,20], and Tlr5 [21]. Increased levels of circulating FFAs also lead to macrophage activation through Tlrs resulting in the production of downstream proinflammatory cytokines [22]. A HFD enriched with saturated fatty acids facilitates inflammatory response and affects insulin sensitivity [23,24]. Studies have confirmed that non-functional Tlr2 [25,26,27] and Tlr4 [28,29,30,31,32] improved insulin sensitivity and lowered inflammation in DIO. Furthermore, the deletion of interleukin 1 receptor-associated kinase 1 gene (*Irak1*) improves glucose tolerance primarily by increasing insulin sensitivity in mice put under a HFD [33].

MicroRNAs (miRNAs) are highly conserved small non-coding RNA molecules involved in post-transcriptional regulation of protein levels [34]. miRNAs play important roles in the regulation of the innate immune system [35,36,37]. They play a pivotal role in regulating the adipocyte differentiation as well as in the development of obesity and associated fat metabolism [38,39]. Aberrant expression of miRNAs has been observed in adipose tissues in HFD-induced obese mice [40]. For example, increased *miR-143* in the mesenteric fat tissue of HFD-fed mice was found to be involved in the pathophysiology of obesity and contributed to the regulation of gene expression in adipocytes [41,42]. Ectopic expression of *miR-103* in pre-adipocytes has been demonstrated to accelerate adipogenesis [41]. Deregulation of *miR-33* and *miR-122*, the major regulators of lipid metabolism in liver, has been related to obesity and metabolic syndrome [43]. *MiR-34a* inhibits brown fat formation in obesity [44]; the *miR-34a* knockout mice are more susceptible to DIO [45]. The experiment with an adipocyte-specific knockout of *miR-200b/a/429* cluster demonstrated the essential role of the gene cluster in the regulation of metabolic changes induced by HFD in the whole body. [46]. In contrast, an inactive *miR-155* prevents DIO and increases adipogenesis and insulin sensitivity, while limiting inflammation in the adipose tissue [47]. Ablation of *miR-155* also leads to decreased atherosclerosis, increased non-alcoholic fatty liver disease and accumulation of white adipose tissue [48]. The gene *miR-378* is one of the pivot regulatory factors in controlling the expansion of brown adipose tissue [49]. The knockout of *miR-378* and miR-378* in mice resulted in enhanced mitochondrial fatty acid metabolism, elevated oxidative capacity of the target tissues of insulin, and increased resistance to HFD-induced obesity [50].

To treat DIO and its related metabolic disorders, decreasing the intake and increasing the expenditure of energy have been commonly recommended [51]. Calorie restriction is effective in decreasing both body weight and body fat percentage [52]. Switching from HFD to low-fat diet (LFD) can effectively reduce body weight and improve insulin sensitivity [53]. Evidence collected from these inbred mouse strains suggests that the detrimental effects of HFD in metabolism are strain-dependent [54,55]. However, currently there is little information on the effect of LFD on obese mice with inactive Tlr and on the expression and regulation of miRNAs during the process of weight reduction. In this study, we aimed to investigate the effect of LFD on Tlr5, Tlr2, and Tlr4 knockout mice with DIO compared to the standard C57BL/6 mice with DIO.

## 2. Materials and Methods

### 2.1. Animal Experiments

Eight-week-old male mice weighing 20–25g were used in the study. C57BL/6 mice were purchased from BioLasco (Taipei, Taiwan). *Tlr* gene knockout mice-Tlr5^−/−^ (B6.129S1-Tlr5^tm1Flv^/J), Tlr2^−/−^ (B6.129-Tlr2^tm1Kir^/J), Tlr4^−/−^ (C57BL/10ScNJ) were purchased from Jackson Laboratory (Bar Harbor, ME, USA). All these Tlr-knockout mice have a C57BL/6 genetic background and the C57BL/6 mice are suggested to be the most appropriate controls by Jackson Laboratory. The animal experiment was performed after the protocols were approved by the Institutional Animal Care and Use Committee (IACUC) of Kaohsiung Chang Gung Memorial Hospital. AIN-76A is a standard reference diet that is formulated by a committee of the American Institute of Nutrition for rodents throughout the biomedical research community [56]. The C57BL/6 mouse will develop obesity and diabetes if raised on a high-fat diet, and this obesity could be completely reversed by reducing dietary fat [57,58,59]. In this study, 24 mice of each strain were randomly assigned to three subgroups (*n* = 8) as follows: (1) the control in which mice were fed ad libitum the standard diet, AIN-76A (with 11.5% kcal fat) for 12 weeks, (2) the DIO in which mice were induced obesity by feeding ad libitum a HFD with 58% kcal fat (D12331; Research Diets Inc., New Brunswick, NJ, USA) for 12 weeks, and (3) the diet in which mice were induced obesity by feeding ad libitum the same HFD (D12331) for eight weeks and thereafter fed a LFD with 10.5% kcal fat (D12329; Research Diets Inc., New Brunswick, NJ, USA) for four weeks to lose weight. Weight was recorded weekly. At the twelfth week of the experiment, the mice were euthanized, and the epididymal fat pads of each mouse was immediately dissected, weighed, and frozen in liquid nitrogen and stored at −80 °C prior to further analysis. The study was conducted in accordance with the Declaration of Helsinki, and the protocol was approved by the Ethics Committee of Chang Gung Memorial Hospital Center for Laboratory Animals. (Project identification code 2012091002). The housing and feeding of the mice were carried out in a specific pathogen free (SPF) facility accredited by the Association for Assessment and Accreditation of Laboratory Animal Care (AAALAC) and in accordance with the national and institutional guidelines.

### 2.2. Histological Examination

Three 5 µm-thick sections, 50 µm apart, were taken from the same paraffin-embedded fat specimen obtained from each mouse. The sections were stained with hematoxylin and eosin (H&E). The microscopic fields at 200× magnification for each section were photographed and 100 adipose cells in the central field were randomly selected to measure the adipocyte size using Image-Pro Plus image analysis software version 6.0 (Carl Zeiss, Oberkochen, Germany). The size of adipocytes was expressed in terms of square micrometers.

### 2.3. Deep Sequencing of Small RNA

Total RNA was extracted from epididymal fat pads using the mirVana™ miRNA isolation kit (Life Technologies, Grand Island, NY, USA). The purified RNA yield was determined by the absorbance at 260 nm with an SSP-3000 Nanodrop spectrophotometer (Infinigen Biotechnology, Inc., City of Industry, CA, USA), and the quality was evaluated using Bioanalyzer 2100 (Agilent Technologies, Santa Clara, CA, USA). The small RNA samples were sent to Genomics Biotech Co., Ltd. (Genomics, Taiwan) for small RNA cloning and sequencing. The population of miRNAs with a length of 15–30 nucleotides was passively eluted from polyacrylamide gels. The RNA was then precipitated with ethanol (TCI, Taipei, Taiwan) and dissolved in water. Linkers were ligated to the small RNAs and bar-coded cDNAs were prepared using a TruSeq Small RNA Sample Prep Kit (Illumina, San Diego, CA, USA) following the manufacturer’s instructions. Subsequently, adapters were ligated to the end-repaired cDNA fragments using T4 DNA ligase (New England BioLabs, Ipswich, MA, USA) at room temperature for 15 min. Adapter-ligated RNA was reverse-transcribed with SuperScript II Reverse Transcriptase (Invitrogen, Carlsbad, CA, USA), amplified using polymerase chain reaction (PCR), and purified to make a cDNA library with cDNAs approximately 200 bp in size. For each subgroup, the bar-coded PCR products from four mice were pooled to get a quantity more than 2 µg. A total of 12 samples were sent for sequencing using Illumina HiSeq2000 Sequencer (Illumina) with 50 bp single-end read sequencing cycles. The generated next generation sequencing (NGS) data of each sample were analyzed with miRSeq reagent kit v3 (Illumina) [60] with default parameters to quantify the expression of *Mus musculus* miRNAs using miRBase v.21.0 (Illumina).

### 2.4. Determination of Differentially Expressed miRNA

We employed real-time quantitative reverse transcription polymerase chain reaction (RT–qPCR) to determine the expression profiles of sixteen miRNAs selected from NGS analysis. Out of these miRNAs, those which fit the following criteria were considered: (1) there are >500 sequence reads of the miRNA in at least one of the 12 samples (from the three subgroups among the four mouse strains) as shown by the NGS analysis, (2) the miRNA shows a fold change >2.0 or <0.5 with *p* < 0.05 (these miRNAs were considered as differentially expressed in DIO or under diet in a mouse strain). Each RNA sample was reverse transcribed to cDNA by using TaqMan^®^ MicroRNA Reverse Transcription Kit (Applied Biosystems, Foster City, CA, USA) according to the manufacturer’s instructions. For this, the PCR products were mixed with the TaqMan Universal PCR Master Mix (No UNG, PN 4324018, Applied Biosystems, Foster city, CA, USA) and specific miRNA primers from the TaqMan MicroRNA Assays (Applied Biosystems), with the expression of U6 small nuclear RNA as an internal control. The following TaqMan MicroRNA Assays were used in this study: *mmu-miR-101b-3p*, *mmu-miR-103-3p*, *mmu-miR-10a-5p*, *mmu-miR-122-5p*, *mmu-miR-140-3p*, *mmu-miR-145a-5p*, *mmu-miR-192-5p*, *mmu-miR-1a-3p*, *mmu-miR-22-3p*, *mmu-miR-23a-3p*, *mmu-miR-30a-5p*, *mmu-miR-3107-5p*, *mmu-miR-34c-5p*, *mmu-miR-378a-3p*, *mmu-miR-486-5p*, and *mmu-miR-92a-3p*. Each sample for RT–qPCR was run in triplicate by a 7500 real-time PCR system (Applied Biosystems). Expression of a miRNA in each subgroup under each strain was considered differentially regulated if the mean value of all the samples (*n* = 6 for each subgroup) was different from that of its control by more than two fold (*p*-value < 0.05). The induction was expressed as fold change in miRNA expression relative to that in the C57BL/6 control.

### 2.5. Target Prediction and Functional Annotation of Differentially Expressed miRNA

To evaluate functions of differentially expressed miRNAs during dieting, target prediction and functional annotation were performed using the miRSystem database, which is a web-based system that integrates seven well-known miRNA target gene prediction programs: DIANA-microT web server v5.0 (http://www.microrna.gr/webServer), miRanda, miRBridge, PicTar, PITA, rna22, and TargetScan and two experimentally validated databases, TarBase and miRecords (http://mirsystem.cgm.ntu.edu.tw/) [61]. The miRSystem can identify the biological functions/pathways regulated by miRNAs based on the enriched functions of their target genes [61]. The analysis parameters in miRSystem were set as following: (1) hit frequency = 5, (2) observed to expected (O/E) ratio = 2, (3) minimal size of genes annotated by ontology term for testing >50, and (4) pathways matched should be from Kyoto Encyclopedia of Genes and Genomes (KEGG) database [62]. The ratio of the values from the experimental group to those from the control group for queried miRNAs were used to make a weighted pathway ranking score to identify the enriched biological functions.

### 2.6. Statistical Analysis

All experimental data were expressed as the mean ± standard error of the mean. Using SPSS 22 (IBM, Armonk, NY, USA) software, intergroup comparison was performed using analysis of variance (ANOVA) with a Bonferroni post hoc correction to identify significant differences in body weight, weight of fat tissue, and adipocyte size. *p*-values < 0.05 were considered significant.

## 3. Results

### 3.1. Body Weight Reduction by Low-Fat Diet (LFD)

**Figure 1:**
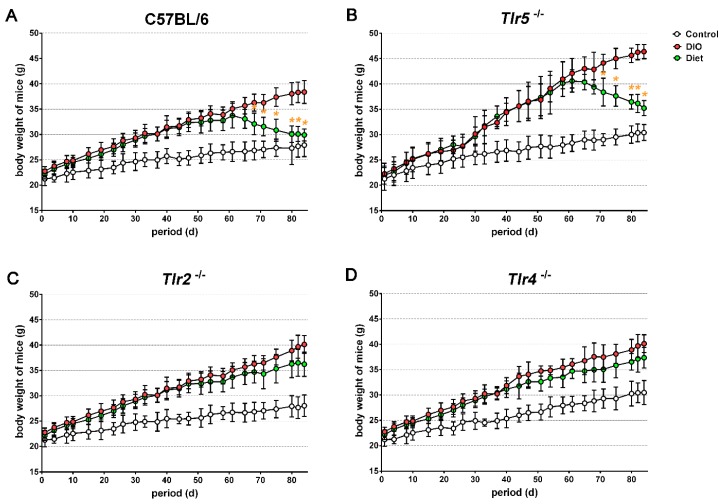
The curve showing the body weights of (**A**) C57BL/6 and (**B**–**D**) toll–like receptors (*Tlr*) knockout mice grouped as control, diet-induced obesity (DIO), and diet. (yellow color), * *p* < 0.05 vs. DIO.

Compared to the regular chow, more body weight was gained by mice of all strains when fed with the HFD. In week 12, the difference in body weight between the DIO and the control groups was found to be 10.8 g in C57BL/6 mice (38.3 ± 2.0 vs. 27.5 ± 1.6 g, Figure 1A), 15.6 g in Tlr5^−/−^ mice (46.1 ± 1.6 vs. 30.5 ± 1.9 g, Figure 1B), 12.1 g in Tlr2^−/−^ mice (40.0 ± 2.2 vs. 27.9 ± 2.4 g, Figure 1C), and 9.7 g in Tlr4^−/−^ mice (40.1 ± 1.9 vs. 30.4 ± 2.5 g, Figure 1D). The Tlr5^−/−^ mice gained significantly more weight than the mice of other strains when put under the HFD (*p* < 0.001). The feeding of LFD significantly reduced the body weight of HFD-fed C57BL/6 and Tlr5^−/−^ mice as observed in week 3 under LFD. After four weeks of feeding LFD, there was reduction in the body weight by 8.4 g in C57BL/6 mice and 9.9 g in Tlr5^−/−^ mice. In contrast, the feeding of LFD for four weeks did not result in a significant body weight between the DIO and diet groups throughout the experiment in Tlr2^−/−^ and Tlr4^−/−^ mice, albeit the body weight of the diet group of these strains was less than that in the DIO group.

### 3.2. Adiposity in Response to Body Weight Reduction

**Figure 2:**
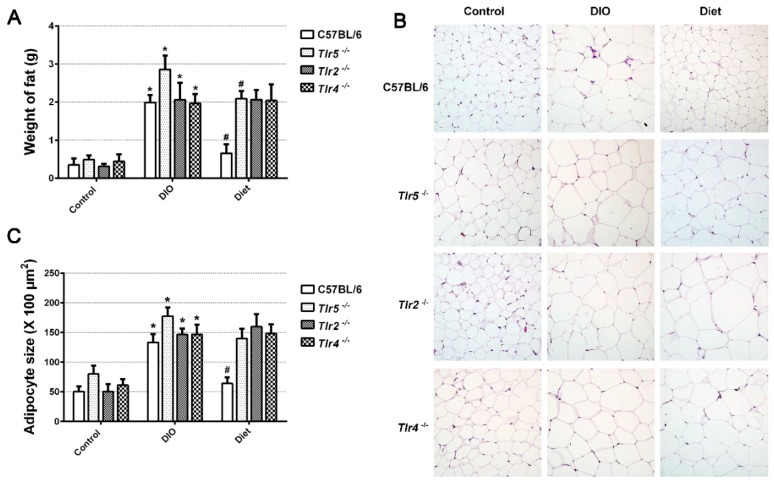
(**A**) The weight of epididymal fat pad, (**B**) hematoxylin and eosin stained 5 μm section of paraffin-embedded epididymal fat pad of C57BL/6 and *Tlr* knockout mice in the control and DIO groups, obtained on the twelfth week, (**C**) size of adipocytes in C57BL/6 and *Tlr* knockout mice. * *p* < 0.05 vs. control. **^#^** *p* < 0.05 vs. DIO.

Compared to those fed with the regular chow, the mice of all strains fed with HFD for 12 weeks showed an increase in the average epididymal fat mass, with average 1.6 g increase in C57BL/6, 2.4 g in Tlr5^−/−^, 1.7 g in Tlr2^−/−^, and 1.5 g in Tlr4^−/−^ (Figure 2A). Feeding of LFD for four weeks significantly decreased the epididymal fat mass in C57BL/6 and Tlr5^−/−^ mice. However, switching to LFD did not result in a significant reduction of epididymal fat mass in Tlr2^−/−^ and Tlr4^−/−^ mice. Histological examination of the epididymal fat (Figure 2B) revealed that the adipocytes in DIO were significantly larger than those in the control in all strains (Figure 2C). After switching to LFD for four weeks, there was significant reduction in the size of adipocytes in C57BL/6, but not in the Tlr5^−/−^, Tlr2^−/−^, and Tlr4^−/−^ mice. Adipocyte hypertrophy was still found in the epididymal fat pads in all the *Tlr* knockout mice.

### 3.3. Expression of miRNAs in the Epididymal Fat

**Figure 3:**
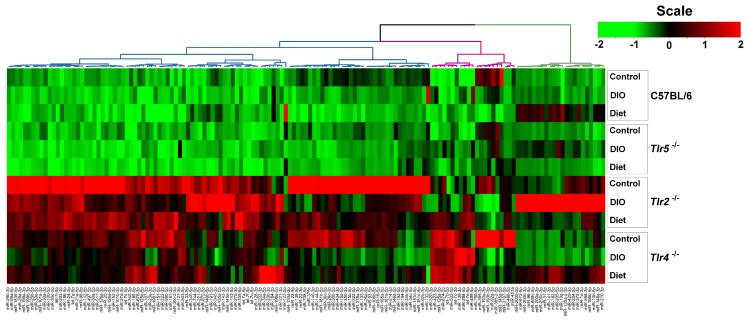
Unsupervised clustering of the dysregulated miRNAs of C57BL/6 and *Tlr* knockout mice in the control and DIO groups, obtained on the twelfth week.

**Figure 4:**
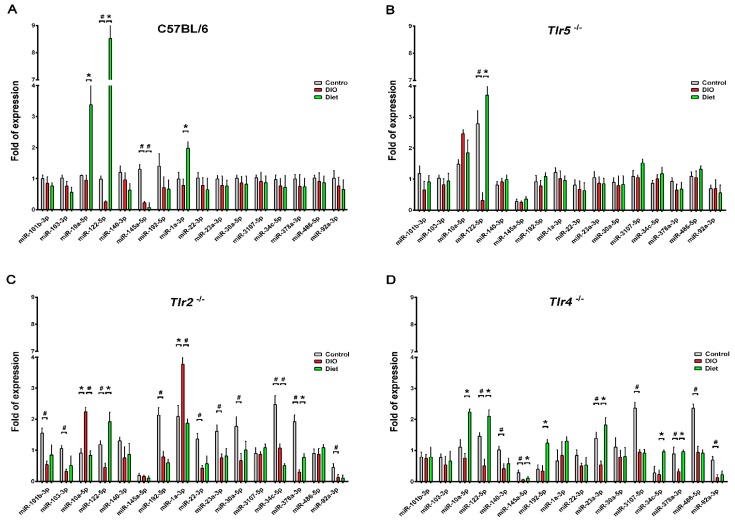
Expression of dysregulated miRNAs in real-time quantitative reverse transcription polymerase chain reaction (RT–qPCR) in the DIO group of C57BL/6 (**A**), Tlr5^−/−^ (**B**), Tlr2^−/−^ (**C**), and Tlr4^−/−^ (**D**) mice after weight reduction. * increase expression with *p* < 0.05 ^#^ decrease expression with *p* < 0.05.

To profile the miRNAs expressed in the epididymal fat pads during DIO, 12 libraries generated from pooling small RNAs were applied to NGS analysis. The number and proportions of the categories of small RNAs found are given in the supplemental Table S1. After filtering the low-quality sequences, empty adaptors and single-read sequences, the selected reads from these libraries mapped well to the mice genome, amounting to 77.04% and 83.72% of the total reads, with miRNAs comprising from 72.03% to 83.72% of the total reads. There were 147 differentially expressed, known miRNAs disclosed by the NGS with >500 sequence reads, found in at least one of the samples. The clustering of these dysregulated miRNAs revealed that the abundance of these miRNAs were widely varied especially in the epididymal fat pads of Tlr2^−/−^ and Tlr4^−/−^ mice. However, the miRNAs found dysregulated in Tlr2^−/−^ and Tlr4^−/−^ mice were different from those in C57BL/6 and Tlr5^−/−^ strains (Figure 3). Sixteen significantly dysregulated miRNAs revealed from different strains were further analyzed by RT–qPCR. The results revealed that in the epididymal fat pads of C57BL/6 mice there was a significantly reduced expression of *miR-122-5p* and *miR-145a-5p* after HFD feeding, and after four weeks of feeding LFD, further significant downregulation of *miR-145a-5p* but upregulation of *miR-10a-5p*, *miR-122-5p*, and *miR-1a-3p*, with around 3.3, 8.5 to 2.0-fold expression respectively resulted (Figure 4A). In Tlr5^−/−^ mice, there was only one dysregulated miRNA. Expression of *miR-122-5p* was significantly decreased after HFD feeding but increased under diet (Figure 4B); a similar pattern was found in all strains-C57BL/6, Tlr5^−/−^, Tlr2^−/−^ (Figure 4C), and Tlr4^−/−^ (Figure 4D). In Tlr2^−/−^ and Tlr4^−/−^ mice, the HFD feeding resulted in more dysregulated miRNAs in the epididymal fat pads. As shown in Figure 4C, the feeding of Tlr2^−/−^ mice with LFD for four weeks resulted in downregulation of *miR-10a-5p* (0.4-fold), *miR-1a-3p* (0.5-fold), and *miR-34c-5p* (0.5-fold) but upregulation of *miR-122-5p* (2.9-fold) and *miR-378a-3p* (2.4-fold). In addition, the feeding of Tlr4^−/−^ mice (Figure 4D) with LFD for four weeks resulted in the upregulation of *miR-10a-5p* (2.1-fold), *miR-122-5p* (3.7-fold), *miR-145a-5p* (2.5-fold), *miR-192a-5p* (2.6-fold), *miR-23a-3p* (3.6-fold), and *miR-378a-3p* (2.3-fold).

### 3.4. Target Prediction and Functional Annotation of Dysregulated miRNAs

**Figure 5:**
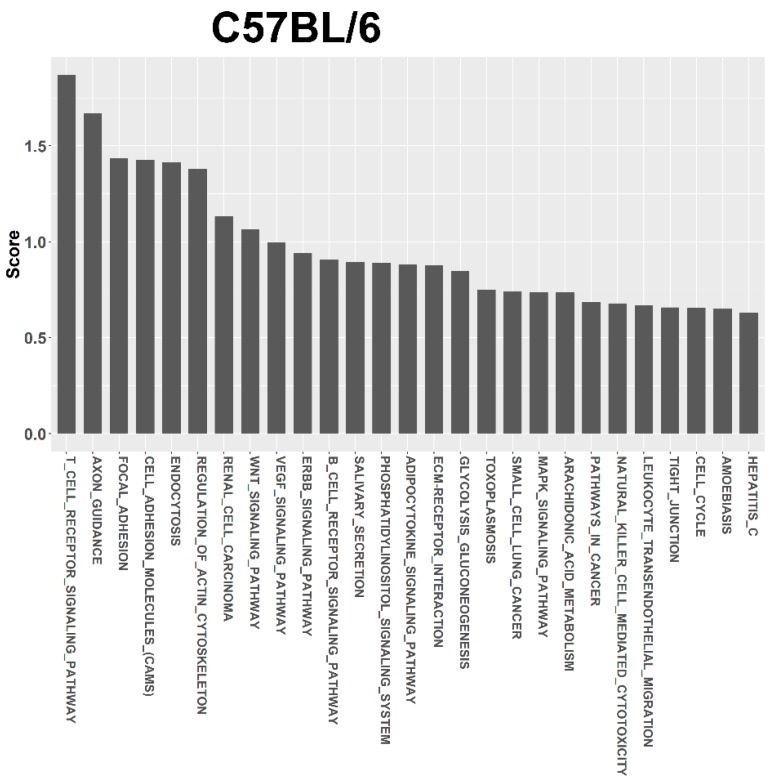
Pathway ranking summery of the genes by the dysregulated miRNAs from miRSystem database during weight reduction in C57BL/6 mice. These pathways were annotated according to KEGG.

**Figure 6:**
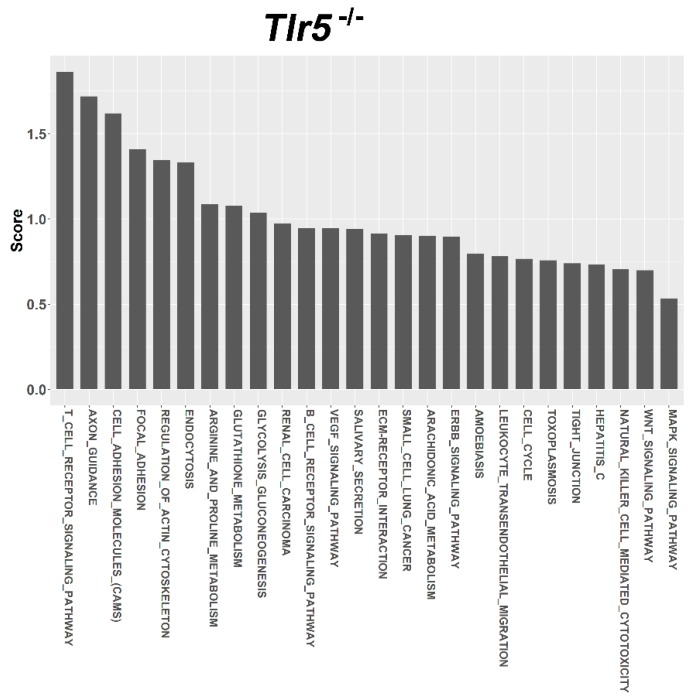
Pathway ranking summery of the genes by the dysregulated miRNAs from miRSystem database during weight reduction in Tlr5^−/−^ mice. These pathways were annotated according to KEGG.

**Figure 7:**
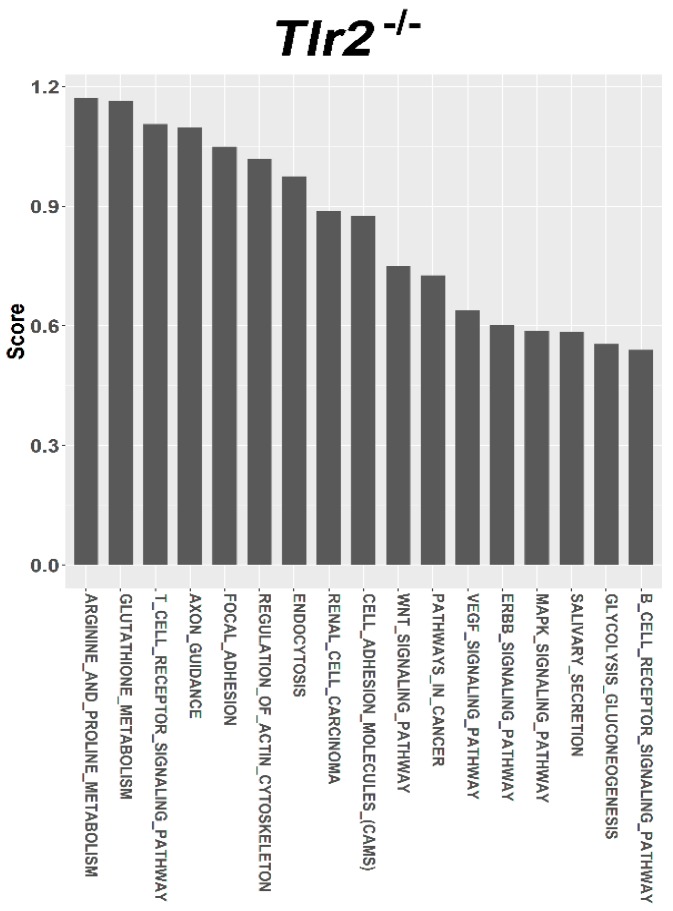
Pathway ranking summery of the genes by the dysregulated miRNAs from miRSystem database during weight reduction in Tlr2^−/−^ mice. These pathways were annotated according to KEGG.

**Figure 8:**
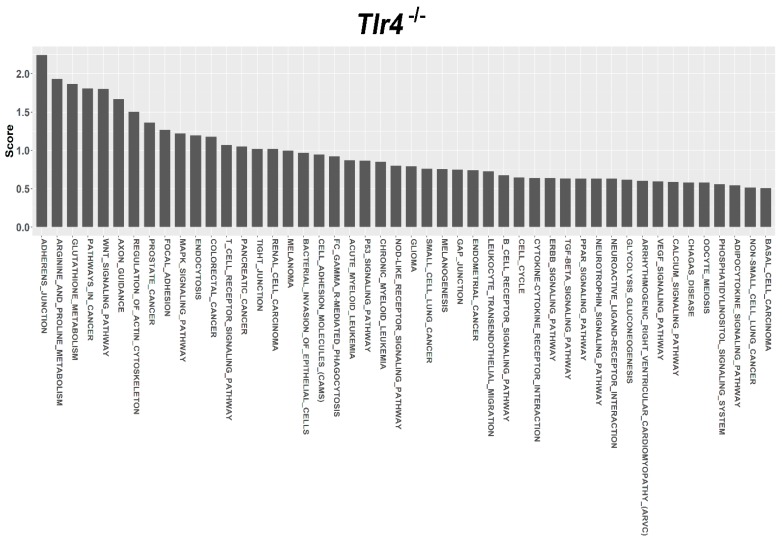
Pathway ranking summery of the genes by the dysregulated miRNAs from miRSystem database during weight reduction in Tlr4^−/−^ mice. These pathways were annotated according to KEGG.

For target prediction and functional annotation, the interaction between major susceptible genes and characteristic dysregulated miRNAs was determined using the miRSystem. In the LFD-fed C57BL/6, Tlr5^−/−^, Tlr2^−/−^, and Tlr4^−/−^ mice, results indicated that 301, 37, 340, and 447 putative targets, respectively, were regulated by these dysregulated miRNAs (Supplemental Table S2). Among these targets, O/E ratios of 45, 35, 33, and 32 targets respectively were more than 2.0. Functional annotation showed that these targets were significantly enriched in 27, 26, 17, 48 pathways that scored more than 0.5 (Supplemental Table S3) in the LFD-fed C57BL/6 (Figure 5), Tlr5^−/−^ (Figure 6), Tlr2^−/−^ (Figure 7), and Tlr4^−/−^ (Figure 8) mice, respectively. In C57BL/6 mice, the top 10 KEGG pathways involved in weight reduction included (1) T-cell receptor signaling pathway, (2) axon guidance, (3) focal adhesion, (4) cell adhesion molecules, (4) endocytosis, (5) regulation of actin cytoskeleton, (6) renal cell carcinoma, (7) WNT signaling pathway, (8) VEGF signaling pathway, and (10) ERBB signaling pathway (Table 1). All these ten pathways were also found associated with LFD feeding in the Tlr5^−/−^, Tlr2^−/−^, and Tlr4^−/−^ strains. The top six pathways were the same in C57BL/6 and Tlr5^−/−^ mice and most of the regulated pathways in C57BL/6 were also found in Tlr5^−/−^, Tlr2^−/−^, and Tlr4^−/−^ strains. Notably, among those top ten pathways, the pathways of arginine and proline metabolism as well as glutathione metabolism were not found involved in C57BL/6 mice but was shown to be the top one and two pathways in Tlr2^−/−^ mice, top two and three pathways in Tlr4^−/−^ mice, and top seven and eight pathways in Tlr5^−/−^ mice. In addition, the pathway associated with adherens junction was involved in Tlr4^−/−^ mice but not in the other strains.

## 4. Discussion

From the metabolic point of view, the visceral fat tissues are highly active tissues [63,64]. With increased production of adipokines and cytokines, the excess adipose tissues are mostly responsible for the manifestation of associated metabolic complications [65]. It is believed that the benefits of weight loss are attributed to the decreased secretion of inflammatory cytokines by the adipocytes [66,67,68]. Additionally, as the big adipocytes are more prone to rupture, they become a focus of inflammation, as indicated by the positive correlation between adipocyte size and inflammatory cytokines reported in a study [69]. In this study, switching to a LFD significantly reduced the body weight and epididymal fat mass of HFD-fed C57BL/6 and Tlr5^−/−^ mice but not of the Tlr2^−/−^ and Tlr4^−/−^ mice. Weight-reduction with LFD resulted in a different effect on the size of adipocytes in epididymal fat pads in C57BL/6 mice compared to the mice lacking *Tlr5*, *Tlr2*, and *Tlr4* genes. 

The study analyzed 16 significantly dysregulated miRNAs from NGS analysis with RT–qPCR and revealed that feeding LFD upregulated *miR-10a-5p*, *miR-122-5p*, and *miR-1a-3p* in the epididymal fat tissue of C57BL/6 mice. In Tlr5^−/−^ mice, only *miR-122-5p* was upregulated. In contrast, in Tlr2^−/−^ and Tlr4^−/−^ mice, LFD feeding resulted in more aberrant expression of miRNAs. In this study, the abundance of these miRNAs widely varied among the experimental samples from different mouse strains; the miRNA expression pattern in the epididymal fat of Tlr2^−/−^ and Tlr4^−/−^ mice was quite different from that in C57BL/6 and Tlr5^−/−^ mice. Among these miRNAs, *miR-122* has recently been revealed as one of the key regulators of lipid metabolism [43,70]. Elevated circulating *miR-122* was found be to positively associated with obesity and insulin resistance in young human adults [71] and in a murine model of obesity [72]. As noted, the expression of *miR-122-5p* was significantly decreased after HFD feeding but increased under the diet in mice of all strains. *MiR-378a* regulates energy homeostasis and enhances adipogenesis by targeting mitogen-activated protein kinase 1 [73,74] and acts as a pivot regulatory factor for controlling the expansion of the brown adipose tissue [49]. Inhibition of *miR-378a* expression attenuated lipolysis and decreased the expression of lipolytic genes, while over-expression of *miR-378a* increased the expression of lipolytic regulators [75]. In addition, the expression of *miR-103-3p* and *miR-1a* has been reported to regulate the genes related to glucose and fatty acid metabolism [76] and *miR-23a-3p*, to be associated with obesity and insulin resistance [77]. However, no related reports regarding the *miR-10a-5p*, *miR-145a-5p*, *miR-192a-5p*, and *miR-34c* with obesity or adipogenesis had been reported earlier.

Regardless of the varied miRNA expression in mice of different strains in association with feeding LFD, target prediction and functional annotation by the miRSystem database showed that targets of these miRNAs were significantly enriched in similar KEGG pathways in all these mice. The top 10 KEGG pathways involved in weight reduction in C57BL/6 mice were also involved in the Tlr5^−/−^, Tlr2^−/−^, and Tlr4^−/−^ mice. The top six pathways were same between C57BL/6 and Tlr5^−/−^ mice and most of the regulated pathways in C57BL/6 could also be found in that of Tlr5^−/−^, Tlr2^−/−^, and Tlr4^−/−^ mice. These results implied that there were common pathways involved in the physiological response during weight reduction regardless of the mouse strains. Interestingly, gene sets for the pathways of arginine and proline metabolism as well as glutathione metabolism were found involved in the *Tlr* knockout mice but not in C57BL/6 mice. This leads to the question whether these gene sets play an important role in the manifestation of different effects in C57BL/6 and *Tlr* knockout mice during the weight reduction with LFD. The association of arginine and glutathione with obesity has been widely studied. As the nitrogenous precursor of nitric oxide (NO), L-arginine regulates multiple metabolic pathways involved in adipogenesis [78]. Available evidence shows that physiological levels of arginine and NO decrease fat synthesis and promote the oxidation of fat in a tissue-specific manner [79]. L-arginine has been reported to prevent body fat accumulation in DIO by regulating pancreatic β-cell physiology [80]. Supplementation of arginine reduces adiposity and improves glucose tolerance in obese rodents and humans [81]. In genetically obese rats, additive arginine promotes gut hormone release with reduced intake of the food [82] and decreases the white fat mass [83]. Furthermore, the link between obesity and oxidative stress has long been recognized. Excessive accumulation of reactive oxygen species in adipose tissue has been implicated in the development of insulin resistance and type 2 diabetes [84]. Glutathione represents the major thiol-disulfide redox system within all cell types [85]. Plasma concentration of glutathione decreases in obesity [86]. In contrast, diet restriction affected gene sets involved in glutathione and fatty acid metabolism [87]. Specifically the depletion of glutathione in adipocytes exhibited restricted adipose expansion associated with increased ectopic lipid accumulation and deteriorated insulin sensitivity [88]. Depletion of glutathione also induces fat remodeling [89], enhances insulin sensitivity, and prevents obesity by HFD [90].

TLRs are powerful molecular regulators by which the immune system may sense the environment and protect the host from invading pathogens [91] or endogenous threats [92]. Tlr2 recognizes a variety of bacterial components from Gram positive bacteria [93] and zymosan in the cell membrane fungus [94]. Tlr4 is activated by the bacterial cell wall components such as lipopolysaccharide [39]. Activation of Tlr2- and Tlr4- signaling pathways in host cells were shown to play a key role in cytokine secretion to eliminate the infectious organisms [91]. Tlr5 recognizes bacterial flagellin and is a transmembrane protein that is highly expressed in the intestinal mucosa [11,12]. Activation of Tlr5 signaling leads to a proinflammatory response with the production of IL-17 and IL-22 that in turn promote the clearance of pathogens [95]. In addition, in pathological conditions, endogenous ligands are passively and actively released by the cells and, interacting with specific TLRs (Tlr2 and Tlr4 in particular), they can regulate the inflammatory response in the tissue [92]. Evidence for the connection between the overall gut microbial composition and obesity had been reported [96,97]. Microbiota plays an important role in the complex network of obesity and metabolic disorders. HFD and bacteria interact to promote early inflammatory changes in the intestine and this contributes to the susceptibility to obesity [98]. Development of obesity in mice, due to genetic susceptibility or induction by diet is associated with dramatic changes in the composition and metabolic function of the microbiota [99]. The interesting question of whether there is an association between the microbiota and the different phenotype manifestations in the *Tlr* knockout mice during weight reduction, was not explored in this study. Further investigation on this may provide more information about the different responses of C57BL/6 and *Tlr* knockout mice to weight reduction by LFD.

## 5. Conclusions

In this study, we demonstrated that feeding of LFD leads to significant body weight reduction in DIO mice established by feeding with the HFD in the mice strains of C57BL/6 and Tlr5^−/−^, but not in the mice strains of Tlr2^−/−^ and Tlr4^−/−^. Significant reduction in the size of adipocytes of epididymal fat was only found in C57BL/6, but not in Tlr5^−/−^, Tlr2^−/−^, and Tlr4^−/−^ mice. The dysregulated miRNAs in Tlr2^−/−^ and Tlr4^−/−^ mice were different from those in C57BL/6 and Tlr5^−/−^ strains. Among those miRNA-regulated pathways, arginine and proline metabolism as well as glutathione metabolism may have important roles in the *Tlr* knockout mice rather than in C57BL/6 mice.

## Figures and Tables

**Table 1:** The top 10 Kyoto Encyclopedia of Genes and Genomes (KEGG) pathways involved in weight reduction in C57BL/6, Tlr5, Tlr2, Tlr4 mice.

Term	Term ID	Total Genes of the Term	Union Targets in the Term	Mirnas in the Term	Score
**C57BL/6**					
T CELL RECEPTOR SIGNALING PATHWAY	4660	109	7	3	1.869
AXON GUIDANCE	4360	131	10	4	1.671
FOCAL ADHESION	4510	197	11	3	1.436
CELL ADHESION MOLECULES (CAMS)	4514	149	4	3	1.428
ENDOCYTOSIS	4144	219	12	4	1.416
REGULATION OF ACTIN CYTOSKELETON	4810	215	11	4	1.379
RENAL CELL CARCINOMA	5211	71	7	2	1.133
WNT SIGNALING PATHWAY	4310	153	9	4	1.065
VEGF SIGNALING PATHWAY	4370	76	4	3	0.998
ERBB SIGNALING PATHWAY	4012	87	5	3	0.941
**TLR5-KO**					
T CELL RECEPTOR SIGNALING PATHWAY	4660	109	2	1	1.864
AXON GUIDANCE	4360	131	2	1	1.719
CELL ADHESION MOLECULES (CAMS)	4514	149	2	1	1.62
FOCAL ADHESION	4510	197	2	1	1.41
REGULATION OF ACTIN CYTOSKELETON	4810	215	2	1	1.346
ENDOCYTOSIS	4144	219	2	1	1.333
ARGININE AND PROLINE METABOLISM	330	53	1	1	1.087
GLUTATHIONE METABOLISM	480	54	1	1	1.079
GLYCOLYSIS GLUCONEOGENESIS	10	60	1	1	1.038
RENAL CELL CARCINOMA	5211	71	1	1	0.973
**TLR2-KO**					
ARGININE AND PROLINE METABOLISM	330	53	2	2	1.171
GLUTATHIONE METABOLISM	480	54	2	2	1.164
T CELL RECEPTOR SIGNALING PATHWAY	4660	109	8	4	1.106
AXON GUIDANCE	4360	131	9	4	1.098
FOCAL ADHESION	4510	197	13	3	1.049
REGULATION OF ACTIN CYTOSKELETON	4810	215	14	4	1.018
ENDOCYTOSIS	4144	219	11	4	0.975
RENAL CELL CARCINOMA	5211	71	8	3	0.888
CELL ADHESION MOLECULES (CAMS)	4514	149	7	4	0.875
WNT SIGNALING PATHWAY	4310	153	10	4	0.749
**TLR4-KO**					
ADHERENS JUNCTION	4520	74	12	4	2.242
ARGININE AND PROLINE METABOLISM	330	53	2	2	1.931
GLUTATHIONE METABOLISM	480	54	2	2	1.864
PATHWAYS IN CANCER	5200	323	21	4	1.808
WNT SIGNALING PATHWAY	4310	153	14	5	1.802
AXON GUIDANCE	4360	131	11	5	1.669
REGULATION OF ACTIN CYTOSKELETON	4810	215	15	5	1.504
PROSTATE CANCER	5215	89	10	3	1.363
FOCAL ADHESION	4510	197	12	4	1.265
MAPK SIGNALING PATHWAY	4010	271	16	5	1.22

## Supplementary Materials

The following are available online at http://www.mdpi.com/2072-6643/10/10/1464/s1, Table S1: The number and proportions of the categories of small RNAs identified from the next generation sequencing, Table S2: The putative targets regulated by the dysregulated miRNAs in the LFD-fed mice, Table S3: Significantly enriched pathways that regulate these targets in the functional annotation.
