# Supplementary material for: Effect of Low-Fat Diet in Obese Mice Lacking Toll-like Receptors
Source: Nutrients. 2018 Oct 9;10(10):1464. doi: 10.3390/nu10101464 (PMC6213519; doi:10.3390/nu10101464)
Supplement: Supplementary file 1 [file nutrients-10-01464-s001.zip › nutrients-352441-SI.pdf]

Supplemental Table S1. The number and proportions of the categories of small RNAs identified from the next generation sequencing.

**B6 CTRL**					**B6 DIO**					**B6 Diet**				
**Category**	unique sRNAs	%	total sRNAs	%	**Category**	unique sRNAs	%	total sRNAs	%	**Category**	unique sRNAs	%	total sRNAs	%
**Total**	141269	100%	5930301	100%	**Total**	240478	100%	5927735	100%	**Total**	86415	100%	5898832	100%
**Mapping to genome**	76583	54.21%	4736082	79.86%	**Mapping to genome**	150523	62.59%	4681507	78.98%	**Mapping to genome**	43950	50.86%	4815750	81.64%
**exon_antisense**	601	0.43%	944	0.02%	**exon_antisense**	1162	0.48%	1827	0.03%	**exon_antisense**	496	0.57%	628	0.01%
**exon_sense**	17161	12.15%	19652	0.33%	**exon_sense**	24657	10.25%	34234	0.58%	**exon_sense**	11213	12.98%	12493	0.21%
**intron_antisense**	1470	1.04%	1828	0.03%	**intron_antisense**	3439	1.43%	5951	0.10%	**intron_antisense**	1409	1.63%	1687	0.03%
**intron_sense**	5831	4.13%	8300	0.14%	**intron_sense**	5893	2.45%	8839	0.15%	**intron_sense**	3626	4.20%	7538	0.13%
**miRNA**	2827	2.00%	4531022	76.40%	**miRNA**	2884	1.20%	4269688	72.03%	**miRNA**	2855	3.30%	4722821	80.06%
**rRNA**	11531	8.16%	91390	1.54%	**rRNA**	8843	3.68%	40904	0.69%	**rRNA**	5925	6.86%	29374	0.50%
**repeat**	9937	7.03%	15039	0.25%	**repeat**	23098	9.61%	51467	0.87%	**repeat**	4757	5.50%	7285	0.12%
**scRNA**	121	0.09%	21453	0.36%	**scRNA**	121	0.05%	6779	0.11%	**scRNA**	101	0.12%	6495	0.11%
**snRNA**	1112	0.79%	4854	0.08%	**snRNA**	799	0.33%	3657	0.06%	**snRNA**	723	0.84%	4211	0.07%
**snoRNA**	1065	0.75%	4076	0.07%	**snoRNA**	799	0.33%	3049	0.05%	**snoRNA**	770	0.89%	2633	0.04%
**srpRNA**	147	0.10%	292	0.00%	**srpRNA**	82	0.03%	167	0.00%	**srpRNA**	64	0.07%	102	0.00%
**tRNA**	7093	5.02%	60630	1.02%	**tRNA**	4983	2.07%	30820	0.52%	**tRNA**	4000	4.63%	26489	0.45%
**unann**	82373	58.31%	1170821	19.74%	**unann**	163718	68.08%	1470353	24.80%	**unann**	50476	58.41%	1077076	18.26%

**TLR5KO CTRL**					**TLR5KO DIO**					**TLR5KO Diet**				
**Category**	unique sRNAs	%	total sRNAs	%	**Category**	unique sRNAs	%	total sRNAs	%	**Category**	unique sRNAs	%	total sRNAs	%
**Total**	174192	100%	5901707	100%	**Total**	161343	100%	5890537	100%	**Total**	195072	100%	5901509	100%
**Mapping to genome**	79123	45.42%	4623954	78.35%	**Mapping to genome**	89281	55.34%	4703871	79.85%	**Mapping to genome**	101087	51.82%	4546575	77.04%
**exon_antisense**	462	0.27%	628	0.01%	**exon_antisense**	529	0.33%	631	0.01%	**exon_antisense**	555	0.28%	783	0.01%
**exon_sense**	25951	14.90%	28186	0.48%	**exon_sense**	37044	22.96%	41831	0.71%	**exon_sense**	29650	15.20%	33140	0.56%
**intron_antisense**	1383	0.79%	1602	0.03%	**intron_antisense**	1424	0.88%	1618	0.03%	**intron_antisense**	1544	0.79%	2023	0.03%
**intron_sense**	8471	4.86%	11906	0.20%	**intron_sense**	13763	8.53%	17989	0.31%	**intron_sense**	7578	3.88%	8798	0.15%
**miRNA**	2870	1.65%	4343798	73.60%	**miRNA**	2926	1.81%	4533273	76.96%	**miRNA**	2796	1.43%	4196062	71.10%
**rRNA**	20609	11.83%	196147	3.32%	**rRNA**	10796	6.69%	55826	0.95%	**rRNA**	11612	5.95%	61176	1.04%
**repeat**	8857	5.08%	12740	0.22%	**repeat**	10166	6.30%	13097	0.22%	**repeat**	12022	6.16%	18297	0.31%

**scRNA**	175	0.10%	17525	0.30%	**scRNA**	178	0.11%	11233	0.19%	**scRNA**	160	0.08%	18107	0.31%
**snRNA**	1743	1.00%	6619	0.11%	**snRNA**	1489	0.92%	6414	0.11%	**snRNA**	1261	0.65%	4936	0.08%
**snoRNA**	1348	0.77%	5243	0.09%	**snoRNA**	1445	0.90%	6618	0.11%	**snoRNA**	1203	0.62%	5175	0.09%
**srpRNA**	298	0.17%	1105	0.02%	**srpRNA**	106	0.07%	272	0.00%	**srpRNA**	127	0.07%	315	0.01%
**tRNA**	13468	7.73%	137147	2.32%	**tRNA**	6815	4.22%	42082	0.71%	**tRNA**	12243	6.28%	253634	4.30%
**unann**	88557	50.84%	1139061	19.30%	**unann**	74662	46.28%	1159653	19.69%	**unann**	114321	58.60%	1299063	22.01%
**TLR2KO CTRL**					**TLR2KO DIO**					**TLR2KO Diet**				
**Category**	unique sRNAs	%	total sRNAs	%	**Category**	unique sRNAs	%	total sRNAs	%	**Category**	unique sRNAs	%	total sRNAs	%
**Total**	218993	100%	11834622	100%	**Total**	178892	100%	13068165	100%	**Total**	166532	100%	12129404	100%
**Mapping to genome**	108599	49.59%	9681121	81.80%	**Mapping to genome**	97092	54.27%	10940191	83.72%	**Mapping to genome**	84767	50.90%	9994121	82.40%
**exon_antisense**	738	0.34%	892	0.01%	**exon_ antisense**	1012	0.57%	1125	0.01%	**exon_ antisense**	684	0.41%	829	0.01%
**exon_sense**	44515	20.33%	52101	0.44%	**exon_sense**	41121	22.99%	46605	0.36%	**exon_sense**	32985	19.81%	37965	0.31%
**intron_antisense**	1812	0.83%	2405	0.02%	**intron_ antisense**	2582	1.44%	3386	0.03%	**intron_ antisense**	1850	1.11%	2230	0.02%
**intron_sense**	18033	8.23%	28612	0.24%	**intron_ sense**	11115	6.21%	15336	0.12%	**intron_ sense**	12871	7.73%	19423	0.16%
**miRNA**	3940	1.80%	9333779	78.87%	**miRNA**	3769	2.11%	10717234	82.01%	**miRNA**	3644	2.19%	9789754	80.71%
**rRNA**	15819	7.22%	165085	1.39%	**rRNA**	9899	5.53%	62092	0.48%	**rRNA**	8632	5.18%	53974	0.44%
**repeat**	12842	5.86%	21485	0.18%	**repeat**	10746	6.01%	15812	0.12%	**repeat**	10595	6.36%	14778	0.12%
**scRNA**	200	0.09%	48843	0.41%	**scRNA**	141	0.08%	10501	0.08%	**scRNA**	164	0.10%	20945	0.17%
**snRNA**	1558	0.71%	9305	0.08%	**snRNA**	1264	0.71%	6658	0.05%	**snRNA**	1216	0.73%	6537	0.05%
**snoRNA**	1370	0.63%	5411	0.05%	**snoRNA**	1016	0.57%	3349	0.03%	**snoRNA**	1049	0.63%	3719	0.03%
**srpRNA**	241	0.11%	779	0.01%	**srpRNA**	147	0.08%	298	0.00%	**srpRNA**	86	0.05%	234	0.00%
**tRNA**	12250	5.59%	135838	1.15%	**tRNA**	8125	4.54%	100681	0.77%	**tRNA**	6472	3.89%	74657	0.62%
**unann**	105675	48.25%	2030087	17.15%	**unann**	87955	49.17%	2085088	15.96%	**unann**	86284	51.81%	2104359	17.35%
**TLR4KO CTRL**					**TLR4KO DIO**					**TLR4KO Diet**				
**Category**	unique sRNAs	%	total sRNAs	%	**Category**	unique sRNAs	%	total sRNAs	%	**Category**	unique sRNAs	%	total sRNAs	%
**Total**	164982	100%	11814561	100%	**Total**	109268	100%	10000689	100%	**Total**	205689	100%	10736921	100%
**Mapping to genome**	71840	43.54%	9390339	79.48%	**Mapping to genome**	46018	42.11%	8173299	81.73%	**Mapping to genome**	111688	54.30%	8737687	81.38%
**exon_antisense**	486	0.29%	720	0.01%	**exon_ antisense**	560	0.0051	833	0.0001	**exon_ antisense**	1208	0.59%	1621	0.02%
**exon_sense**	23805	14.43%	26912	0.23%	**exon_sense**	17773	16.27%	20038	0.20%	**exon_sense**	26566	12.92%	31441	0.29%
**intron_antisense**	1391	0.84%	1803	0.02%	**intron_**	1670	1.53%	1940	0.02%	**intron_**	3217	1.56%	4158	0.04%

**intron_sense**	9913	6.01%	12985	0.11%	**antisense intron_ sense**	4897	4.48%	10318	0.10%	**antisense intron_ sense**	9029	4.39%	16906	0.16%
**miRNA**	3539	2.15%	9192905	77.81%	**miRNA**	3141	2.87%	8075567	80.75%	**miRNA**	3797	1.85%	8489118	79.06%
**rRNA**	13749	8.33%	79695	0.67%	**rRNA**	6955	6.37%	38624	0.39%	**rRNA**	10094	4.91%	59485	0.55%
**repeat**	8009	4.85%	11637	0.10%	**repeat**	4330	3.96%	6482	0.06%	**repeat**	14912	7.25%	26451	0.25%
**scRNA**	157	0.10%	29952	0.25%	**scRNA**	97	0.09%	3174	0.03%	**scRNA**	149	0.07%	9813	0.09%
**snRNA**	1410	0.85%	7732	0.07%	**snRNA**	727	0.67%	4143	0.04%	**snRNA**	1150	0.56%	6900	0.06%
**snoRNA**	1494	0.91%	6684	0.06%	**snoRNA**	753	0.69%	2271	0.02%	**snoRNA**	1070	0.52%	3165	0.03%
**srpRNA**	122	0.07%	236	0.00%	**srpRNA**	58	0.05%	184	0.00%	**srpRNA**	100	0.05%	241	0.00%
**tRNA**	7538	4.57%	54943	0.47%	**tRNA**	4178	3.82%	27930	0.28%	**tRNA**	5721	2.78%	45490	0.42%
**unann**	93369	56.59%	2388357	20.22%	**unann**	64129	58.69%	1809185	18.09%	**unann**	128676	62.56%	2042132	19.02%

Supplemental Table S2. The putative targets regulated by the dysregulated miRNAs in the LFD-fed mice.

Supplemental Table S2A. C57BL6.

TARGET_GENE	GENE_DESCRIPTION	KEGG	OBSERVED_MIRNA	OE
NRDG3		0	1	128.25
HSPA1B	heat shock protein 1B	6	1	16.0313
GJA3	gap junction protein, alpha 3	0	1	12.825
ANXA2	annexin A2	0	1	6.75001
ANP32B	acidic (leucine-rich) nuclear phosphoprotein 32 family, member B	0	1	6.41249
ALDOA	aldolase A, fructose-bisphosphate	4	1	5.34375
JUND	Jun proto-oncogene related gene d	1	1	5.13
RTKN	rhotekin	0	1	4.93269
SRSF9	serine/arginine-rich splicing factor 9	1	1	4.93269
CDK9	cyclin-dependent kinase 9 (CDC2-related kinase)	0	1	4.75
PHAX	phosphorylated adaptor for RNA export	1	1	4.275
HSPD1	heat shock protein 1 (chaperonin)	2	1	3.77206
SNX15	sorting nexin 15	0	1	3.77206
TRPV6	transient receptor potential cation channel, subfamily V, member 6	1	1	3.77206
DAZAP1	DAZ associated protein 1	0	1	3.5625
HSP90B1	heat shock protein 90, beta (Grp94), member 1	4	1	3.5625
SMAP1	stromal membrane-associated protein 1	1	2	3.5625
TMED3	transmembrane emp24 domain containing 3	0	1	3.5625
TMSB4X	thymosin, beta 4, X chromosome	1	1	3.5625
IRX5	Iroquois related homeobox 5 (Drosophila)	0	1	3.46621
SLC25A1	solute carrier family 25 (mitochondrial carrier, citrate transporter), member 1	0	1	3.375

HFE2	hemochromatosis type 2 (juvenile) (human homolog)	0	1	3.28846
TMEM178	transmembrane protein 178	0	2	3.28846
ACTB	actin, beta	12	1	3.12805
GIT1	G protein-coupled receptor kinase-interactor 1	2	2	3.01765
KTN1	kinectin 1	0	1	2.98256
TRAPPC3	trafficking protein particle complex 3	0	1	2.78804
MAPK3	mitogen-activated protein kinase 3	49	1	2.72872
ATXN2	ataxin 2	0	1	2.67187
CALM2	calmodulin 2	15	1	2.67187
BRPF1	bromodomain and PHD finger containing, 1	0	1	2.61735
SPATS2	spermatogenesis associated, serine-rich 2	0	1	2.565
RRBP1	ribosome binding protein 1	1	1	2.46633
SNX2	sorting nexin 2	0	1	2.46633
CACHD1	cache domain containing 1	0	1	2.41981
HAND2	heart and neural crest derivatives expressed transcript 2	0	1	2.41981
STC2	stanniocalcin 2	0	1	2.41981
PDGFA	platelet derived growth factor, alpha	10	1	2.29017
SLC35A4	solute carrier family 35, member A4	0	1	2.29017
FSCN1	fascin homolog 1, actin bundling protein (Strongylocentrotus purpuratus)	0	1	2.21122
GPX7	glutathione peroxidase 7	2	1	2.17372
MAF1	MAF1 homolog (S. cerevisiae)	0	1	2.1375
ZFP423	zinc finger protein 423	0	1	2.10247
CCNL1	cyclin L1	0	1	2.06854
PLCL2	phospholipase C-like 2	0	1	2.00391
DHX15	DEAH (Asp-Glu-Ala-His) box polypeptide 15	1	1	1.97307
RGS7	regulator of G protein signaling 7	0	1	1.94318
MAB21L1	mab-21-like 1 (C. elegans)	0	1	1.91418
FXR2	fragile X mental retardation, autosomal homolog 2	0	1	1.88602
HDAC4	histone deacetylase 4	0	1	1.88602
NCOR2	nuclear receptor co-repressor 2	1	1	1.88602
NONO	non-POU-domain-containing, octamer binding protein	0	1	1.85869
CDC42BPB	CDC42 binding protein kinase beta	0	1	1.83215
CNN3	calponin 3, acidic	0	1	1.83215
GABARAPL2	gamma-aminobutyric acid (GABA) A receptor-associated protein-like 2	1	1	1.83215
TBP	TATA box binding protein	2	1	1.7331
SEC22B	SEC22 vesicle trafficking protein homolog B (S. cerevisiae)	2	1	1.6875
CITED2	Cbp/p300-interacting transactivator, with Glu/Asp-rich carboxy-terminal domain, 2	0	2	1.62342
SP2	Sp2 transcription factor	0	1	1.62342

AP3D1	adaptor-related protein complex 3, delta 1 subunit	1	1	1.60313
FAM135A	family with sequence similarity 135, member A	0	1	1.60313
NOTCH3	Notch gene homolog 3 (Drosophila)	2	1	1.60313
ARSJ	arylsulfatase J	0	1	1.56402
YTHDF2	YTH domain family 2	0	1	1.56402
KIF2A	kinesin family member 2A	0	1	1.50882
NPEPPS	aminopeptidase puromycin sensitive	0	1	1.50882
PDCD4	programmed cell death 4	0	1	1.50882
FZD7	frizzled homolog 7 (Drosophila)	4	2	1.5
FNDC3A	fibronectin type III domain containing 3A	0	2	1.49128
KCNIP3	Kv channel interacting protein 3, calsenilin	0	1	1.49128
OTX2	orthodenticle homolog 2 (Drosophila)	0	1	1.45739
WDR1	WD repeat domain 1	0	1	1.45739
CAPRIN1	cell cycle associated protein 1	0	2	1.43296
FAM108C	family with sequence similarity 108, member C	0	1	1.39402
SLITRK4	SLIT and NTRK-like family, member 4	0	1	1.39402
USP46	ubiquitin specific peptidase 46	0	2	1.39402
MATR3	matrin 3	0	1	1.36436
BDNF	brain derived neurotrophic factor	3	2	1.35
MEST	mesoderm specific transcript	0	1	1.35
ANXA4	annexin A4	0	1	1.33593
GTF2H1	general transcription factor II H, polypeptide 1	2	1	1.33593
CD320	CD320 antigen	0	1	1.32216
FAM108B	family with sequence similarity 108, member B	0	1	1.32216
HLTF	helicase-like transcription factor	0	1	1.30867
MPZ	myelin protein zero	1	1	1.30867
TRA2B	transformer 2 beta homolog (Drosophila)	1	1	1.30867
BCKDK	branched chain ketoacid dehydrogenase kinase	0	1	1.29546
HMGN1	high mobility group nucleosomal binding domain 1	0	1	1.29546
POLDIP3	polymerase (DNA-directed), delta interacting protein 3	0	1	1.29546
TRIM2	tripartite motif-containing 2	0	3	1.29111
MED1	mediator complex subunit 1	0	2	1.28895
PAQR9	progestin and adipoQ receptor family member IX	0	1	1.2825
ANGPT2	angiopoietin 2	0	1	1.2698
CAP1	CAP, adenylate cyclase-associated protein 1 (yeast)	0	1	1.25736
FUNDC2	FUN14 domain containing 2	0	1	1.25736
REV3L	REV3-like, catalytic subunit of DNA polymerase zeta RAD54 like (S. cerevisiae)	1	1	1.25736
SLITRK6	SLIT and NTRK-like family, member 6	0	1	1.25736

TNFRSF11B	tumor necrosis factor receptor superfamily, member 11b (osteoprotegerin)	1	1	1.25736
EPB4.1L5	erythrocyte protein band 4.1-like 5	0	1	1.24514
HIAT1	hippocampus abundant gene transcript 1	0	1	1.23317
PDCD10	programmed cell death 10	0	1	1.2099
FLI1	Friend leukemia integration 1	0	1	1.1986
ARCN1	archain 1	0	1	1.1875
GALNT1	UDP-N-acetyl-alpha-D-galactosamine:polypeptide N-acetylgalactosaminyltransferase 1	2	1	1.1875
HNRNPK	heterogeneous nuclear ribonucleoprotein K	1	1	1.1875
PICALM	phosphatidylinositol binding clathrin assembly protein	0	1	1.1875
CCDC97	coiled-coil domain containing 97	0	1	1.1766
GLIS2	GLIS family zinc finger 2	0	1	1.16591
ATP6V1B2	ATPase, H+ transporting, lysosomal V1 subunit B2	4	1	1.15541
TCFAP2C	transcription factor AP-2, gamma	0	1	1.15541
P4HA1	procollagen-proline, 2-oxoglutarate 4-dioxygenase (proline 4-hydroxylase), alpha 1 polypeptide	2	1	1.14509
CNNM4	cyclin M4	0	1	1.13496
DUSP6	dual specificity phosphatase 6	1	1	1.13496
PTPLAD1	protein tyrosine phosphatase-like A domain containing 1	0	1	1.13496
GRK6	G protein-coupled receptor kinase 6	2	1	1.125
OCLN	occludin	4	1	1.125
PHC2	polyhomeotic-like 2 (Drosophila)	0	1	1.125
SPOP	speckle-type POZ protein	0	1	1.11522
RNF216	ring finger protein 216	0	1	1.1056
ANKRD13C	ankyrin repeat domain 13c	0	1	1.09615
CLTC	clathrin, heavy polypeptide (Hc)	4	1	1.09615
SLC25A25	solute carrier family 25 (mitochondrial carrier, phosphate carrier), member 25	0	1	1.08687
SNAP25	synaptosomal-associated protein 25	1	1	1.08687
SRSF3	serine/arginine-rich splicing factor 3	1	1	1.08687
HIC2	hypermethylated in cancer 2	0	2	1.08686
SRSF1	serine/arginine-rich splicing factor 1	1	2	1.08228
KDM2B	lysine (K)-specific demethylase 2B	0	1	1.07773
TM9SF4	transmembrane 9 superfamily protein member 4	0	1	1.05992
EDN1	endothelin 1	4	1	1.05123
FIGN	fidgetin	0	1	1.04268
INO80	INO80 homolog (S. cerevisiae)	0	1	1.04268
SOX9	SRY-box containing gene 9	0	1	1.04268
SULF1	sulfatase 1	0	1	1.03428
TNPO2	transportin 2 (importin 3, karyopherin beta 2b)	0	1	1.03428
CTNNBIP1	catenin beta interacting protein 1	1	1	1.026

KLF11	Kruppel-like factor 11	0	1	1.026
ADAR	adenosine deaminase, RNA-specific	1	1	1.01786
CPEB1	cytoplasmic polyadenylation element binding protein 1	3	1	1.00984
GAS2L1	growth arrest-specific 2 like 1	0	1	1.00984
RIT2	Ras-like without CAAX 2	0	1	1.00984
SENP2	SUMO/sentrin specific peptidase 2	2	1	1.00984
FNDC3B	fibronectin type III domain containing 3B	0	2	1.00195
CYTH1	cytohesin 1	0	1	0.994186
LARP4B	La ribonucleoprotein domain family, member 4B	0	1	0.994186
TWF1	twinfilin, actin-binding protein, homolog 1 (Drosophila)	0	1	0.994186
BCL6	B-cell leukemia/lymphoma 6	0	1	0.98654
CS	citrate synthase	3	1	0.98654
EBF2	early B-cell factor 2	0	1	0.979006
FGF14	fibroblast growth factor 14	4	1	0.971591
UBA6	ubiquitin-like modifier activating enzyme 6	1	1	0.964287
TBR1	T-box brain gene 1	0	1	0.95
DAB2	disabled homolog 2 (Drosophila)	1	1	0.943015
TMEM50B	transmembrane protein 50B	0	1	0.943015
VAMP2	vesicle-associated membrane protein 2	3	1	0.943015
ZFP36L1	zinc finger protein 36, C3H type-like 1	0	1	0.943015
EIF4E	eukaryotic translation initiation factor 4E	3	1	0.93613
KANK4	KN motif and ankyrin repeat domains 4	0	1	0.93613
LENG8	leukocyte receptor cluster (LRC) member 8	0	1	0.929347
GJA1	gap junction protein, alpha 1	2	1	0.916073
SDC1	syndecan 1	3	1	0.909574
CBL	Casitas B-lineage lymphoma	9	1	0.903169
GATA6	GATA binding protein 6	0	1	0.903169
ANKRD28	ankyrin repeat domain 28	0	1	0.896855
MAP3K3	mitogen-activated protein kinase kinase kinase 3	3	1	0.896855
SLC25A36	solute carrier family 25, member 36	0	1	0.890624
DPYSL2	dihydropyrimidinase-like 2	1	1	0.878426
FOSB	FBJ osteosarcoma oncogene B	0	1	0.872448
ADD3	adducin 3 (gamma)	0	1	0.854999
GNPNAT1	glucosamine-phosphate N-acetyltransferase 1	1	1	0.854999
RAD21	RAD21 homolog (S. pombe)	1	1	0.854999
SERP1	stress-associated endoplasmic reticulum protein 1	0	1	0.854999
CREM	cAMP responsive element modulator	0	1	0.849338
JARID2	jumonji, AT rich interactive domain 2	0	1	0.849338

MDFIC	MyoD family inhibitor domain containing	0	1	0.849338
MAP3K12	mitogen-activated protein kinase kinase kinase 12	1	1	0.843751
TIAM1	T-cell lymphoma invasion and metastasis 1	2	1	0.843751
HOXA1	homeobox A1	0	1	0.838234
TMEM55B	transmembrane protein 55b	0	1	0.838234
ATP6V1A	ATPase, H+ transporting, lysosomal V1 subunit A	4	1	0.832792
FKBP5	FK506 binding protein 5	0	1	0.832792
SMARCC1	SWI/SNF related, matrix associated, actin dependent regulator of chromatin, subfamily c, member 1	0	1	0.82742
CDC42	cell division cycle 42 homolog (S. cerevisiae)	18	1	0.822114
SPRED1	sprouty protein with EVH-1 domain 1, related sequence	1	1	0.816879
ZMYND11	zinc finger, MYND domain containing 11	0	1	0.811709
CTTNBP2NL	CTTNBP2 N-terminal like	0	1	0.801562
UTRN	utrophin	0	1	0.801562
HIVEP2	human immunodeficiency virus type I enhancer binding protein 2	0	1	0.796584
GRB10	growth factor receptor bound protein 10	0	1	0.782012
NCK2	non-catalytic region of tyrosine kinase adaptor protein 2	3	1	0.777274
RCAN2	regulator of calcineurin 2	0	1	0.777274
SMAD3	MAD homolog 3 (Drosophila)	10	1	0.777274
YES1	Yamaguchi sarcoma viral (v-yes) oncogene homolog 1	2	1	0.777274
YWHAZ	tyrosine 3-monooxygenase/tryptophan 5-monooxygenase activation protein, zeta polypeptide	3	1	0.77259
API5	apoptosis inhibitor 5	0	1	0.763394
LANCL1	LanC (bacterial lantibiotic synthetase component C)-like 1	0	1	0.763394
SFRP1	secreted frizzled-related protein 1	1	1	0.758875
UST	uronyl-2-sulfotransferase	1	1	0.754412
IVNS1ABP	influenza virus NS1A binding protein	0	1	0.750001
ZIC4	zinc finger protein of the cerebellum 4	0	1	0.745639
CPLX2	complexin 2	0	1	0.741329
RNGTT	RNA guanylyltransferase and 5'-phosphatase	0	1	0.732856
UBN1	ubinuclein 1	0	1	0.732856
VEGFA	vascular endothelial growth factor A	8	1	0.728693
CFL2	cofilin 2, muscle	3	1	0.724577
LASS6	LAG1 homolog, ceramide synthase 6	0	1	0.724577
LDLRAD3	low density lipoprotein receptor class A domain containing 3	0	1	0.720505
NFAT5	nuclear factor of activated T-cells 5	6	2	0.718488
CAMK2G	calcium/calmodulin-dependent protein kinase II gamma	11	1	0.708563
TSPYL4	TSPY-like 4	0	1	0.708563
MMD	monocyte to macrophage differentiation-associated	0	1	0.70467
MYLK	myosin, light polypeptide kinase	5	1	0.70467

PEA15A	phosphoprotein enriched in astrocytes 15A	0	1	0.70467
CLCN3	chloride channel 3	0	1	0.70082
MEF2A	myocyte enhancer factor 2A	0	1	0.70082
MXD1	MAX dimerization protein 1	0	1	0.70082
BCL7A	B-cell CLL/lymphoma 7A	0	1	0.697012
MTAP1A	microtubule-associated protein 1 A	0	1	0.697012
RNF138	ring finger protein 138	0	1	0.697012
SEC62	SEC62 homolog (S. cerevisiae)	2	1	0.697012
TIMP3	tissue inhibitor of metalloproteinase 3	0	1	0.697012
D3BWG0562E	DNA segment, Chr 3, Brigham & Women's Genetics 0562 expressed	0	1	0.689516
CALM1	calmodulin 1	15	1	0.68218
CD2AP	CD2-associated protein	1	1	0.68218
AZIN1	antizyme inhibitor 1	0	1	0.678572
CTNND1	catenin (cadherin associated protein), delta 1	2	1	0.678572
MAP3K7	mitogen-activated protein kinase kinase kinase 7	9	1	0.657692
NUAK1	NUAK family, SNF1-like kinase, 1	0	1	0.657692
ARHGAP24	Rho GTPase activating protein 24	0	1	0.654337
CTDSPL	CTD (carboxy-terminal domain, RNA polymerase II, polypeptide A) small phosphatase-like	0	1	0.654337
TPPP	tubulin polymerization promoting protein	0	1	0.654337
CLIC4	chloride intracellular channel 4 (mitochondrial)	0	1	0.651015
NCL	nucleolin	0	1	0.651015
SLC39A10	solute carrier family 39 (zinc transporter), member 10	0	1	0.651015
MNT	max binding protein	0	1	0.647727
THBS1	thrombospondin 1	7	1	0.647727
ZFP367	zinc finger protein 367	0	1	0.647727
VEZF1	vascular endothelial zinc finger 1	0	1	0.644473
ABR	active BCR-related gene	0	1	0.641249
PTPN1	protein tyrosine phosphatase, non-receptor type 1	2	1	0.641249
ASH1L	ash1 (absent, small, or homeotic)-like (Drosophila)	2	1	0.638059
DACH1	dachshund 1 (Drosophila)	0	1	0.638059
ACVR1B	activin A receptor, type 1B	1	1	0.634901
G3BP2	GTPase activating protein (SH3 domain) binding protein 2	0	1	0.631773
ANKIB1	ankyrin repeat and IBR domain containing 1	0	1	0.628676
ERLIN1	ER lipid raft associated 1	0	1	0.628676
DAAM1	dishevelled associated activator of morphogenesis 1	1	1	0.62561
PAK3	p21 protein (Cdc42/Rac)-activated kinase 3	6	1	0.622573
MET	met proto-oncogene	10	1	0.613637
DDX17	DEAD (Asp-Glu-Ala-Asp) box polypeptide 17	0	1	0.610714

MMGT1	membrane magnesium transporter 1	0	1	0.610714
COL4A3	collagen, type IV, alpha 3	0	1	0.604953
LIN7C	lin-7 homolog C (C. elegans)	0	1	0.604953
SEMA6A	sema domain, transmembrane domain (TM), and cytoplasmic domain, (semaphorin) 6A	1	1	0.604953
KLHL18	kelch-like 18 (Drosophila)	0	1	0.599299
BSN	bassoon	0	1	0.596512
ACVR2A	activin receptor IIA	2	1	0.593749
ARID2	AT rich interactive domain 2 (ARID, RFX-like)	0	1	0.593749
HS6ST2	heparan sulfate 6-O-sulfotransferase 2	1	1	0.593749
PTGFR	prostaglandin F receptor	2	1	0.593749
RSBN1	rosbin, round spermatid basic protein 1	0	1	0.591014
ACSL4	acyl-CoA synthetase long-chain family member 4	5	1	0.580317
FOXO1	forkhead box O1	3	1	0.580317
ELAVL2	ELAV (embryonic lethal, abnormal vision, Drosophila)-like 2 (Hu antigen B)	0	1	0.575112
MBTD1	mbt domain containing 1	0	1	0.572545
MBNL1	muscleblind-like 1 (Drosophila)	0	1	0.570001
BAZ2B	bromodomain adjacent to zinc finger domain, 2B	0	1	0.564978
LAMC1	laminin, gamma 1	7	1	0.564978
FNBP1L	formin binding protein 1-like	0	1	0.560043
NDRG3	N-myc downstream regulated gene 3	0	1	0.557609
SYT1	synaptotagmin I	0	1	0.557609
RAB14	RAB14, member RAS oncogene family	0	1	0.552801
SLC7A2	solute carrier family 7 (cationic amino acid transporter, y+ system), member 2	0	1	0.550429
IGF1	insulin-like growth factor 1	13	1	0.548077
ACAP2	ArfGAP with coiled-coil, ankyrin repeat and PH domains 2	1	1	0.54114
WIPF2	WAS/WASL interacting protein family, member 2	0	1	0.54114
ANKFY1	ankyrin repeat and FYVE domain containing 1	0	1	0.538865
MAF	avian musculoaponeurotic fibrosarcoma (v-maf) AS42 oncogene homolog	0	1	0.529959
RAP2A	RAS related protein 2a	0	1	0.523469
FOXP2	forkhead box P2	0	1	0.521342
RYBP	RING1 and YY1 binding protein	0	1	0.517137
RNF38	ring finger protein 38	0	1	0.51506
ESRRG	estrogen-related receptor gamma	0	1	0.510956
FUBP1	far upstream element (FUSE) binding protein 1	0	1	0.506917
BCL2L11	BCL2-like 11 (apoptosis facilitator)	0	1	0.500977
NEGR1	neuronal growth regulator 1	1	1	0.499027
SEMA6D	sema domain, transmembrane domain (TM), and cytoplasmic domain, (semaphorin) 6D	1	1	0.499027
CDK14	cyclin-dependent kinase 14	0	1	0.497093

PAFAH1B1	platelet-activating factor acetylhydrolase, isoform 1b, subunit 1	2	1	0.489504
CAMK1D	calcium/calmodulin-dependent protein kinase ID	0	1	0.483962
ETS1	E26 avian leukemia oncogene 1, 5' domain	2	1	0.482142
FBXO28	F-box protein 28	0	1	0.482142
FAM126A	family with sequence similarity 126, member A	0	1	0.480337
MYO5A	myosin VA	0	1	0.480337
RICTOR	RPTOR independent companion of MTOR, complex 2	1	1	0.475
TGFBR2	transforming growth factor, beta receptor II	10	1	0.473247
PLAGL2	pleiomorphic adenoma gene-like 2	0	1	0.461331
FRS2	fibroblast growth factor receptor substrate 2	1	1	0.437713
SOX11	SRY-box containing gene 11	0	1	0.434746
MTF1	metal response element binding transcription factor 1	0	1	0.424669
GABRB2	gamma-aminobutyric acid (GABA) A receptor, subunit beta 2	1	1	0.409745
ZFP148	zinc finger protein 148	0	1	0.368534

Supplemental Table S2B. TLR5.

TARGET_GENE	GENE_DESCRIPTION	KEGG	OBSERVED_MIRNA	OE
NRDG3		0	1	512.999
ALDOA	aldolase A, fructose-bisphosphate	4	1	21.375
TRPV6	transient receptor potential cation channel, subfamily V, member 6	1	1	15.0882
TMED3	transmembrane emp24 domain containing 3	0	1	14.25
HFE2	hemochromatosis type 2 (juvenile) (human homolog)	0	1	13.1538
BRPF1	bromodomain and PHD finger containing, 1	0	1	10.4694
SLC35A4	solute carrier family 35, member A4	0	1	9.1607
GPX7	glutathione peroxidase 7	2	1	8.6949
MAF1	MAF1 homolog (S. cerevisiae)	0	1	8.55
CDC42BPB	CDC42 binding protein kinase beta	0	1	7.32858
GIT1	G protein-coupled receptor kinase-interactor 1	2	1	6.03529
NPEPPS	aminopeptidase puromycin sensitive	0	1	6.03529
CD320	CD320 antigen	0	1	5.28865
BCKDK	branched chain ketoacid dehydrogenase kinase	0	1	5.18183
FUNDC2	FUN14 domain containing 2	0	1	5.02942
CCDC97	coiled-coil domain containing 97	0	1	4.70641
P4HA1	procollagen-proline, 2-oxoglutarate 4-dioxygenase (proline 4-hydroxylase), alpha 1 polypeptide	2	1	4.58035
OCLN	occludin	4	1	4.5
ANKRD13C	ankyrin repeat domain 13c	0	1	4.38462

CS	citrate synthase	3	1	3.94616
TBR1	T-box brain gene 1	0	1	3.8
TMEM50B	transmembrane protein 50B	0	1	3.77206
RAD21	RAD21 homolog (S. pombe)	1	1	3.42
MAP3K12	mitogen-activated protein kinase kinase kinase 12	1	1	3.375
FKBP5	FK506 binding protein 5	0	1	3.33117
LASS6	LAG1 homolog, ceramide synthase 6	0	1	2.89831
CLIC4	chloride intracellular channel 4 (mitochondrial)	0	1	2.60406
G3BP2	GTPase activating protein (SH3 domain) binding protein 2	0	1	2.52709
PAK3	p21 protein (Cdc42/Rac)-activated kinase 3	6	1	2.49029
MMGT1	membrane magnesium transporter 1	0	1	2.44286
MBNL1	muscleblind-like 1 (Drosophila)	0	1	2.28
LAMC1	laminin, gamma 1	7	1	2.25991
NDRG3	N-myc downstream regulated gene 3	0	1	2.23044
ACAP2	ArfGAP with coiled-coil, ankyrin repeat and PH domains 2	1	1	2.16456
FOXP2	forkhead box P2	0	1	2.08537
NEGR1	neuronal growth regulator 1	1	1	1.99611
NFAT5	nuclear factor of activated T-cells 5	6	1	1.43698

Supplemental Table S2C. TLR2.

TARGET_GENE	GENE_DESCRIPTION	KEGG	OBSERVED_MIRNA	OE
NRDG3		0	1	102.6
HSPA1B	heat shock protein 1B	6	1	12.825
GJA3	gap junction protein, alpha 3	0	1	10.26
ALDOA	aldolase A, fructose-bisphosphate	4	2	8.55
ANXA2	annexin A2	0	1	5.40001
ANP32B	acidic (leucine-rich) nuclear phosphoprotein 32 family, member B	0	1	5.12999
JUND	Jun proto-oncogene related gene d	1	1	4.104
SRSF9	serine/arginine-rich splicing factor 9	1	1	3.94615
CDK9	cyclin-dependent kinase 9 (CDC2-related kinase)	0	1	3.8
PHAX	phosphorylated adaptor for RNA export	1	1	3.42
HSPD1	heat shock protein 1 (chaperonin)	2	1	3.01765
SNX15	sorting nexin 15	0	1	3.01765
TRPV6	transient receptor potential cation channel, subfamily V, member 6	1	1	3.01765
DAZAP1	DAZ associated protein 1	0	1	2.85
HSP90B1	heat shock protein 90, beta (Grp94), member 1	4	1	2.85
ODC1	ornithine decarboxylase, structural 1	3	1	2.85
SMAP1	stromal membrane-associated protein 1	1	2	2.85

TMED3	transmembrane emp24 domain containing 3	0	1	2.85
TMSB4X	thymosin, beta 4, X chromosome	1	1	2.85
IRX5	Iroquois related homeobox 5 (Drosophila)	0	1	2.77297
SLC25A1	solute carrier family 25 (mitochondrial carrier, citrate transporter), member 1	0	1	2.7
HFE2	hemochromatosis type 2 (juvenile) (human homolog)	0	1	2.63077
GIT1	G protein-coupled receptor kinase-interactor 1	2	2	2.41412
JAKMIP1	janus kinase and microtubule interacting protein 1	0	1	2.38605
KTN1	kinectin 1	0	1	2.38605
DGKZ	diacylglycerol kinase zeta	4	1	2.28
PLOD1	procollagen-lysine, 2-oxoglutarate 5-dioxygenase 1	1	1	2.28
TRAPPC3	trafficking protein particle complex 3	0	1	2.23044
MAPK3	mitogen-activated protein kinase 3	49	1	2.18298
CALM2	calmodulin 2	15	1	2.1375
MTA2	metastasis-associated gene family, member 2	0	1	2.1375
BRPF1	bromodomain and PHD finger containing, 1	0	1	2.09388
RGS17	regulator of G-protein signaling 17	0	1	2.09388
RRBP1	ribosome binding protein 1	1	1	1.97307
SNX2	sorting nexin 2	0	1	1.97307
HAND2	heart and neural crest derivatives expressed transcript 2	0	1	1.93585
STC2	stanniocalcin 2	0	1	1.93585
STRN3	striatin, calmodulin binding protein 3	0	1	1.86546
PDGFA	platelet derived growth factor, alpha	10	1	1.83214
SLC35A4	solute carrier family 35, member A4	0	1	1.83214
GPX7	glutathione peroxidase 7	2	1	1.73898
OSGIN2	oxidative stress induced growth inhibitor family member 2	0	1	1.73898
MAF1	MAF1 homolog (S. cerevisiae)	0	1	1.71
DHX15	DEAH (Asp-Glu-Ala-His) box polypeptide 15	1	1	1.57846
MAB21L1	mab-21-like 1 (C. elegans)	0	1	1.53135
FXR2	fragile X mental retardation, autosomal homolog 2	0	1	1.50882
HCN3	hyperpolarization-activated, cyclic nucleotide-gated K+ 3	0	1	1.50882
HDAC4	histone deacetylase 4	0	1	1.50882
NCOR2	nuclear receptor co-repressor 2	1	1	1.50882
VAMP2	vesicle-associated membrane protein 2	3	2	1.50882
NONO	non-POU-domain-containing, octamer binding protein	0	1	1.48696
CDC42BPB	CDC42 binding protein kinase beta	0	1	1.46572
CNN3	calponin 3, acidic	0	1	1.46572
LDHA	lactate dehydrogenase A	5	1	1.40548
TBP	TATA box binding protein	2	1	1.38648

LEF1	lymphoid enhancer binding factor 1	11	1	1.35
PKP4	plakophilin 4	0	1	1.35
SEC22B	SEC22 vesicle trafficking protein homolog B (S. cerevisiae)	2	1	1.35
ACCN1	amiloride-sensitive cation channel 1, neuronal (degenerin)	1	1	1.33247
NUMBL	numb-like	1	1	1.33247
TMEM178	transmembrane protein 178	0	1	1.31538
PNOC	prepronociceptin	0	1	1.29874
SP2	Sp2 transcription factor	0	1	1.29874
AP3D1	adaptor-related protein complex 3, delta 1 subunit	1	1	1.2825
NOTCH3	Notch gene homolog 3 (Drosophila)	2	1	1.2825
WNT1	wingless-related MMTV integration site 1	5	1	1.2825
ARSJ	arylsulfatase J	0	1	1.25122
B4GALT2	UDP-Gal:betaGlcNAc beta 1,4- galactosyltransferase, polypeptide 2	5	1	1.25122
RALGDS	ral guanine nucleotide dissociation stimulator	3	1	1.25122
KIF2A	kinesin family member 2A	0	1	1.20706
NPEPPS	aminopeptidase puromycin sensitive	0	1	1.20706
KCNIP3	Kv channel interacting protein 3, calsenilin	0	1	1.19303
CPLX2	complexin 2	0	2	1.18613
TLK2	tousled-like kinase 2 (Arabidopsis)	0	1	1.17931
OTX2	orthodenticle homolog 2 (Drosophila)	0	1	1.16591
WDR1	WD repeat domain 1	0	1	1.16591
E2F5	E2F transcription factor 5	2	1	1.15281
PEA15A	phosphoprotein enriched in astrocytes 15A	0	2	1.12747
WASF1	WASP family 1	4	1	1.12747
DBC1	deleted in bladder cancer 1 (human)	0	1	1.11522
EI24	etoposide induced 2.4 mRNA	1	1	1.10322
GPR85	G protein-coupled receptor 85	0	1	1.10322
CACNB3	calcium channel, voltage-dependent, beta 3 subunit	5	1	1.09149
FUT8	fucosyltransferase 8	3	1	1.09149
MATR3	matrin 3	0	1	1.09149
BDNF	brain derived neurotrophic factor	3	2	1.08
SRPR	signal recognition particle receptor ('docking protein')	1	1	1.08
ANXA4	annexin A4	0	1	1.06875
GTF2H1	general transcription factor II H, polypeptide 1	2	1	1.06875
CD320	CD320 antigen	0	1	1.05773
MPZ	myelin protein zero	1	1	1.04694
TRA2B	transformer 2 beta homolog (Drosophila)	1	1	1.04694
BCKDK	branched chain ketoacid dehydrogenase kinase	0	1	1.03637

HMGN1	high mobility group nucleosomal binding domain 1	0	1	1.03637
POLDIP3	polymerase (DNA-directed), delta interacting protein 3	0	1	1.03637
RBM14	RNA binding motif protein 14	0	1	1.03637
MED1	mediator complex subunit 1	0	2	1.03116
ZCCHC17	zinc finger, CCHC domain containing 17	0	1	1.026
TAF5	TAF5 RNA polymerase II, TATA box binding protein (TBP)-associated factor	1	1	1.01584
CAP1	CAP, adenylate cyclase-associated protein 1 (yeast)	0	1	1.00588
FUNDC2	FUN14 domain containing 2	0	1	1.00588
PPP1R10	protein phosphatase 1, regulatory subunit 10	0	1	1.00588
DAAM1	dishevelled associated activator of morphogenesis 1	1	2	1.00098
HIAT1	hippocampus abundant gene transcript 1	0	1	0.986539
MET	met proto-oncogene	10	2	0.981819
SGSM2	small G protein signaling modulator 2	0	1	0.977145
DDX17	DEAD (Asp-Glu-Ala-Asp) box polypeptide 17	0	2	0.977142
PDCD10	programmed cell death 10	0	1	0.967923
ARCN1	archain 1	0	1	0.950001
CNTNAP1	contactin associated protein-like 1	1	1	0.950001
GALNT1	UDP-N-acetyl-alpha-D-galactosamine:polypeptide N-acetylgalactosaminyltransferase 1	2	1	0.950001
HNRNPK	heterogeneous nuclear ribonucleoprotein K	1	1	0.950001
PICALM	phosphatidylinositol binding clathrin assembly protein	0	1	0.950001
CCDC97	coiled-coil domain containing 97	0	1	0.941283
GLIS2	GLIS family zinc finger 2	0	1	0.932727
SDHC	succinate dehydrogenase complex, subunit C, integral membrane protein	6	1	0.932727
ATP6V1B2	ATPase, H+ transporting, lysosomal V1 subunit B2	4	1	0.924325
TCFAP2C	transcription factor AP-2, gamma	0	1	0.924325
ARHGAP1	Rho GTPase activating protein 1	0	1	0.91607
MAP2K1	mitogen-activated protein kinase kinase 1	37	1	0.91607
P4HA1	procollagen-proline, 2-oxoglutarate 4-dioxygenase (proline 4-hydroxylase), alpha 1 polypeptide	2	1	0.91607
SEMA4C	sema domain, immunoglobulin domain (Ig), transmembrane domain (TM) and short cytoplasmic domain, (semaphorin) 4C	1	1	0.91607
AXL	AXL receptor tyrosine kinase	0	1	0.907964
CNNM4	cyclin M4	0	1	0.907964
PTPLAD1	protein tyrosine phosphatase-like A domain containing 1	0	1	0.907964
GRK6	G protein-coupled receptor kinase 6	2	1	0.900001
OCLN	occludin	4	1	0.900001
PHC2	polyhomeotic-like 2 (Drosophila)	0	1	0.900001

SYT1	synaptotagmin I	0	2	0.892174
SLC27A4	solute carrier family 27 (fatty acid transporter), member 4	1	1	0.884482
ANKRD13C	ankyrin repeat domain 13c	0	1	0.876924
CLTC	clathrin, heavy polypeptide (Hc)	4	1	0.876924
SLC25A25	solute carrier family 25 (mitochondrial carrier, phosphate carrier), member 25	0	1	0.869493
SNAP25	synaptosomal-associated protein 25	1	1	0.869493
SRSF3	serine/arginine-rich splicing factor 3	1	1	0.869493
SRSF1	serine/arginine-rich splicing factor 1	1	2	0.865823
ACTR1A	ARP1 actin-related protein 1 homolog A, centractin alpha (yeast)	0	1	0.862184
KDM2B	lysine (K)-specific demethylase 2B	0	1	0.862184
CCNE2	cyclin E2	6	1	0.840983
EDN1	endothelin 1	4	1	0.840983
ACSL1	acyl-CoA synthetase long-chain family member 1	5	1	0.834147
EIF2C4	eukaryotic translation initiation factor 2C, 4	0	1	0.834147
FIGN	fidgetin	0	1	0.834147
SERPINE1	serine (or cysteine) peptidase inhibitor, clade E, member 1	3	1	0.834147
SULF1	sulfatase 1	0	1	0.827421
TNPO2	transportin 2 (importin 3, karyopherin beta 2b)	0	1	0.827421
UBP1	upstream binding protein 1	0	1	0.827421
CTNNBIP1	catenin beta interacting protein 1	1	1	0.820799
KLF11	Kruppel-like factor 11	0	1	0.820799
ADAR	adenosine deaminase, RNA-specific	1	1	0.814286
CPEB1	cytoplasmic polyadenylation element binding protein 1	3	1	0.807875
GAS2L1	growth arrest-specific 2 like 1	0	1	0.807875
RIT2	Ras-like without CAAX 2	0	1	0.807875
GPR64	G protein-coupled receptor 64	0	1	0.801561
CYTH1	cytohesin 1	0	1	0.795349
TWF1	twinfilin, actin-binding protein, homolog 1 (Drosophila)	0	1	0.795349
BCL6	B-cell leukemia/lymphoma 6	0	1	0.789232
CS	citrate synthase	3	1	0.789232
ELMOD1	ELMO domain containing 1	0	1	0.789232
NOTCH1	Notch gene homolog 1 (Drosophila)	3	1	0.789232
EBF2	early B-cell factor 2	0	1	0.783205
FGF14	fibroblast growth factor 14	4	1	0.777273
GRM7	glutamate receptor, metabotropic 7	1	1	0.777273
MYO1C	myosin IC	0	1	0.76567
TBR1	T-box brain gene 1	0	1	0.76
TMEM50B	transmembrane protein 50B	0	1	0.754412

ZFP36L1	zinc finger protein 36, C3H type-like 1	0	1	0.754412
EIF4E	eukaryotic translation initiation factor 4E	3	1	0.748904
KANK4	KN motif and ankyrin repeat domains 4	0	1	0.748904
LGR4	leucine-rich repeat-containing G protein-coupled receptor 4	1	1	0.748904
TMEM55A	transmembrane protein 55A	0	1	0.73813
GJA1	gap junction protein, alpha 1	2	1	0.732858
JAG1	jagged 1	1	1	0.727659
PROSAPIP1	ProSAPiP1 protein	0	1	0.727659
SDC1	syndecan 1	3	1	0.727659
CBL	Casitas B-lineage lymphoma	9	1	0.722536
GATA6	GATA binding protein 6	0	1	0.722536
RIC8B	resistance to inhibitors of cholinesterase 8 homolog B (C. elegans)	0	1	0.722536
ZDHHC17	zinc finger, DHHC domain containing 17	0	1	0.722536
METAP1	methionyl aminopeptidase 1	0	1	0.717484
MYRIP	myosin VIIA and Rab interacting protein	0	1	0.717484
ACBD3	acyl-Coenzyme A binding domain containing 3	0	1	0.702741
FOSB	FBJ osteosarcoma oncogene B	0	1	0.697958
TRIM2	tripartite motif-containing 2	0	2	0.68859
GNPNAT1	glucosamine-phosphate N-acetyltransferase 1	1	1	0.683999
RAD21	RAD21 homolog (S. pombe)	1	1	0.683999
SERP1	stress-associated endoplasmic reticulum protein 1	0	1	0.683999
VCL	vinculin	6	1	0.683999
CNTNAP2	contactin associated protein-like 2	1	1	0.67947
CREM	cAMP responsive element modulator	0	1	0.67947
JARID2	jumonji, AT rich interactive domain 2	0	1	0.67947
MAP3K12	mitogen-activated protein kinase kinase kinase 12	1	1	0.675001
TIAM1	T-cell lymphoma invasion and metastasis 1	2	1	0.675001
GALNT7	UDP-N-acetyl-alpha-D-galactosamine: polypeptide N-acetylgalactosaminyltransferase 7	2	1	0.670587
HOXA1	homeobox A1	0	1	0.670587
RRAGC	Ras-related GTP binding C	0	1	0.670587
TMEM55B	transmembrane protein 55b	0	1	0.670587
ATP6V1A	ATPase, H+ transporting, lysosomal V1 subunit A	4	1	0.666234
FKBP5	FK506 binding protein 5	0	1	0.666234
GOLPH3L	golgi phosphoprotein 3-like	0	1	0.661936
SMARCC1	SWI/SNF related, matrix associated, actin dependent regulator of chromatin, subfamily c, member 1	0	1	0.661936
CDC42	cell division cycle 42 homolog (S. cerevisiae)	18	1	0.657691

PPP2R3A	protein phosphatase 2, regulatory subunit B'', alpha	0	1	0.657691
SEMA4F	sema domain, immunoglobulin domain (Ig), TM domain, and short cytoplasmic domain	1	1	0.657691
BTBD11	BTB (POZ) domain containing 11	0	1	0.653503
SPRED1	sprouty protein with EVH-1 domain 1, related sequence	1	1	0.653503
CITED2	Cbp/p300-interacting transactivator, with Glu/Asp-rich carboxy-terminal domain, 2	0	1	0.649368
ZMYND11	zinc finger, MYND domain containing 11	0	1	0.649368
CTTNBP2NL	CTTNBP2 N-terminal like	0	1	0.64125
UTRN	utrophin	0	1	0.64125
HIVEP2	human immunodeficiency virus type I enhancer binding protein 2	0	1	0.637267
SAR1A	SAR1 gene homolog A (S. cerevisiae)	1	1	0.633334
BRPF3	bromodomain and PHD finger containing, 3	0	1	0.621819
CALCR	calcitonin receptor	1	1	0.621819
NCK2	non-catalytic region of tyrosine kinase adaptor protein 2	3	1	0.621819
RCAN2	regulator of calcineurin 2	0	1	0.621819
YWHAZ	tyrosine 3-monooxygenase/tryptophan 5-monooxygenase activation protein, zeta polypeptide	3	1	0.618072
API5	apoptosis inhibitor 5	0	1	0.610715
LANCL1	LanC (bacterial lantibiotic synthetase component C)-like 1	0	1	0.610715
SFRP1	secreted frizzled-related protein 1	1	1	0.6071
RAB43	RAB43, member RAS oncogene family	0	1	0.603529
UST	uronyl-2-sulfotransferase	1	1	0.603529
FZD7	frizzled homolog 7 (Drosophila)	4	1	0.600001
MPP2	membrane protein, palmitoylated 2 (MAGUK p55 subfamily member 2)	0	1	0.600001
FAM70A	family with sequence similarity 70, member A	0	1	0.596511
FNDC3A	fibronectin type III domain containing 3A	0	1	0.596511
PPP1R16B	protein phosphatase 1, regulatory (inhibitor) subunit 16B	0	1	0.596511
ZIC4	zinc finger protein of the cerebellum 4	0	1	0.596511
RNGTT	RNA guanylyltransferase and 5'-phosphatase	0	1	0.586285
UBN1	ubinuclein 1	0	1	0.586285
VEGFA	vascular endothelial growth factor A	8	1	0.582954
LASS6	LAG1 homolog, ceramide synthase 6	0	1	0.579661
NFAT5	nuclear factor of activated T-cells 5	6	2	0.57479
CAPRIN1	cell cycle associated protein 1	0	1	0.573184
CAMK2G	calcium/calmodulin-dependent protein kinase II gamma	11	1	0.56685
TSPYL4	TSPY-like 4	0	1	0.56685
MMD	monocyte to macrophage differentiation-associated	0	1	0.563736
MYH9	myosin, heavy polypeptide 9, non-muscle	3	1	0.563736

MYLK	myosin, light polypeptide kinase	5	1	0.563736
CLCN3	chloride channel 3	0	1	0.560656
MEF2A	myocyte enhancer factor 2A	0	1	0.560656
MXD1	MAX dimerization protein 1	0	1	0.560656
BCL7A	B-cell CLL/lymphoma 7A	0	1	0.557609
MTAP1A	microtubule-associated protein 1 A	0	1	0.557609
RNF138	ring finger protein 138	0	1	0.557609
SEC62	SEC62 homolog (S. cerevisiae)	2	1	0.557609
TIMP3	tissue inhibitor of metalloproteinase 3	0	1	0.557609
USP46	ubiquitin specific peptidase 46	0	1	0.557609
FOXJ2	forkhead box J2	0	1	0.554594
D3BWG0562E	DNA segment, Chr 3, Brigham & Women's Genetics 0562 expressed	0	1	0.551613
CALM1	calmodulin 1	15	1	0.545744
CD2AP	CD2-associated protein	1	1	0.545744
AZIN1	antizyme inhibitor 1	0	1	0.542857
ITCH	itchy, E3 ubiquitin protein ligase	2	1	0.542857
NRIP3	nuclear receptor interacting protein 3	0	1	0.542857
MAP3K7	mitogen-activated protein kinase kinase kinase 7	9	1	0.526154
CTDSPL	CTD (carboxy-terminal domain, RNA polymerase II, polypeptide A) small phosphatase-like	0	1	0.52347
NAV1	neuron navigator 1	0	1	0.52347
TPPP	tubulin polymerization promoting protein	0	1	0.52347
VAT1	vesicle amine transport protein 1 homolog (T californica)	0	1	0.52347
CLIC4	chloride intracellular channel 4 (mitochondrial)	0	1	0.520812
NCL	nucleolin	0	1	0.520812
SLC39A10	solute carrier family 39 (zinc transporter), member 10	0	1	0.520812
MNT	max binding protein	0	1	0.518182
THBS1	thrombospondin 1	7	1	0.518182
ZFP367	zinc finger protein 367	0	1	0.518182
VEZF1	vascular endothelial zinc finger 1	0	1	0.515578
ABR	active BCR-related gene	0	1	0.512999
PTPN1	protein tyrosine phosphatase, non-receptor type 1	2	1	0.512999
ASH1L	ash1 (absent, small, or homeotic)-like (Drosophila)	2	1	0.510448
G3BP2	GTPase activating protein (SH3 domain) binding protein 2	0	1	0.505418
ANKIB1	ankyrin repeat and IBR domain containing 1	0	1	0.502941
CNTN2	contactin 2	1	1	0.502941
BAZ2A	bromodomain adjacent to zinc finger domain, 2A	0	1	0.500488
PAK3	p21 protein (Cdc42/Rac)-activated kinase 3	6	1	0.498059

GPR158	G protein-coupled receptor 158	0	1	0.495652
MMGT1	membrane magnesium transporter 1	0	1	0.488571
COL4A3	collagen, type IV, alpha 3	0	1	0.483963
LIN7C	lin-7 homolog C (C. elegans)	0	1	0.483963
BSN	bassoon	0	1	0.47721
AKAP6	A kinase (PRKA) anchor protein 6	0	1	0.475
ARID2	AT rich interactive domain 2 (ARID, RFX-like)	0	1	0.475
HS6ST2	heparan sulfate 6-O-sulfotransferase 2	1	1	0.475
ARHGAP26	Rho GTPase activating protein 26	0	1	0.472811
NDST1	N-deacetylase/N-sulfotransferase (heparan glucosaminyl) 1	2	1	0.472811
RSBN1	rosbin, round spermatid basic protein 1	0	1	0.472811
ACSL4	acyl-CoA synthetase long-chain family member 4	5	1	0.464254
FBXO41	F-box protein 41	0	1	0.462162
HTR2C	5-hydroxytryptamine (serotonin) receptor 2C	3	1	0.462162
ELAVL2	ELAV (embryonic lethal, abnormal vision, Drosophila)-like 2 (Hu antigen B)	0	1	0.46009
ANKRD52	ankyrin repeat domain 52	0	1	0.458036
PDGFRA	platelet derived growth factor receptor, alpha polypeptide	11	1	0.458036
MBNL1	muscleblind-like 1 (Drosophila)	0	1	0.456001
BAZ2B	bromodomain adjacent to zinc finger domain, 2B	0	1	0.451982
LAMC1	laminin, gamma 1	7	1	0.451982
ZFHX4	zinc finger homeodomain 4	0	1	0.45
FNBP1L	formin binding protein 1-like	0	1	0.448035
NAA50	N(alpha)-acetyltransferase 50, NatE catalytic subunit	0	1	0.448035
SCN2B	sodium channel, voltage-gated, type II, beta	0	1	0.448035
NDRG3	N-myc downstream regulated gene 3	0	1	0.446087
PHF15	PHD finger protein 15	0	1	0.442241
GMFB	glia maturation factor, beta	0	1	0.440343
SLC7A2	solute carrier family 7 (cationic amino acid transporter, y+ system), member 2	0	1	0.440343
TMCC3	transmembrane and coiled coil domains 3	0	1	0.440343
IGF1	insulin-like growth factor 1	13	1	0.438462
HIC2	hypermethylated in cancer 2	0	1	0.434746
ACAP2	ArfGAP with coiled-coil, ankyrin repeat and PH domains 2	1	1	0.432912
FRMPD4	FERM and PDZ domain containing 4	0	1	0.432912
WIPF2	WAS/WASL interacting protein family, member 2	0	1	0.432912
ANKFY1	ankyrin repeat and FYVE domain containing 1	0	1	0.431092
ASB1	ankyrin repeat and SOCS box-containing 1	0	1	0.420491
PKIA	protein kinase inhibitor, alpha	0	1	0.420491
RAP2A	RAS related protein 2a	0	1	0.418775

FOXP2	forkhead box P2	0	1	0.417073
DCAF7	DDB1 and CUL4 associated factor 7	0	1	0.415385
RYBP	RING1 and YY1 binding protein	0	1	0.413709
RNF38	ring finger protein 38	0	1	0.412048
ESRRG	estrogen-related receptor gamma	0	1	0.408765
FUBP1	far upstream element (FUSE) binding protein 1	0	1	0.405534
BCL2L11	BCL2-like 11 (apoptosis facilitator)	0	1	0.400782
FNDC3B	fibronectin type III domain containing 3B	0	1	0.400782
NEGR1	neuronal growth regulator 1	1	1	0.399222
SEMA6D	sema domain, transmembrane domain (TM), and cytoplasmic domain, (semaphorin) 6D	1	1	0.399222
CDK14	cyclin-dependent kinase 14	0	1	0.397674
PAFAH1B1	platelet-activating factor acetylhydrolase, isoform 1b, subunit 1	2	1	0.391603
ETS1	E26 avian leukemia oncogene 1, 5' domain	2	1	0.385714
RICTOR	RPTOR independent companion of MTOR, complex 2	1	1	0.38
CACNA1E	calcium channel, voltage-dependent, R type, alpha 1E subunit	3	1	0.375824
MLLT3	myeloid/lymphoid or mixed-lineage leukemia (trithorax homolog, Drosophila); translocated to, 3	0	1	0.361268
FRS2	fibroblast growth factor receptor substrate 2	1	1	0.350171
CLOCK	circadian locomotor output cycles kaput	1	1	0.344295
MTF1	metal response element binding transcription factor 1	0	1	0.339735
CAMTA1	calmodulin binding transcription activator 1	0	1	0.3375
FRMD4A	FERM domain containing 4A	0	1	0.3375
GABRB2	gamma-aminobutyric acid (GABA) A receptor, subunit beta 2	1	1	0.327796
DCX	doublecortin	0	1	0.295677
TBL1XR1	transducin (beta)-like 1X-linked receptor 1	1	1	0.294827
ZFP148	zinc finger protein 148	0	1	0.294827
ITSN1	intersectin 1 (SH3 domain protein 1A)	0	1	0.278049
TNRC6B	trinucleotide repeat containing 6b	0	1	0.252709

Supplemental Table S2D. TLR4.

TARGET_GENE	GENE_DESCRIPTION	KEGG	OBSERVED_MIRNA	OE
NRDG3		0	1	102.6
MYH4	myosin, heavy polypeptide 4, skeletal muscle	2	1	14.6572
ALDOA	aldolase A, fructose-bisphosphate	4	2	8.55

SNX15	sorting nexin 15	0	2	6.03529
MYH1	myosin, heavy polypeptide 1, skeletal muscle, adult	2	1	4.88571
CACNA2D3	calcium channel, voltage-dependent, alpha2/delta subunit 3	5	1	4.66364
ZFP579	zinc finger protein 579	0	1	4.46087
RTKN	rhotekin	0	1	3.94615
CCK	cholecystokinin	2	1	3.66429
ATP6V1E1	ATPase, H+ transporting, lysosomal V1 subunit E1	4	1	3.53793
VTI1B	vesicle transport through interaction with t-SNAREs 1B homolog	1	1	3.42
ZFP423	zinc finger protein 423	0	2	3.36395
TRPV6	transient receptor potential cation channel, subfamily V, member 6	1	1	3.01765
AUH	AU RNA binding protein/enoyl-coenzyme A hydratase	2	1	2.93143
DAZAP1	DAZ associated protein 1	0	1	2.85
HSP90B1	heat shock protein 90, beta (Grp94), member 1	4	1	2.85
NEFL	neurofilament, light polypeptide	1	1	2.85
TMED3	transmembrane emp24 domain containing 3	0	1	2.85
PKP4	plakophilin 4	0	2	2.7
SLC25A1	solute carrier family 25 (mitochondrial carrier, citrate transporter), member 1	0	1	2.7
HFE2	hemochromatosis type 2 (juvenile) (human homolog)	0	1	2.63077
ACTB	actin, beta	12	1	2.50244
JAKMIP1	janus kinase and microtubule interacting protein 1	0	1	2.38605
DGKZ	diacylglycerol kinase zeta	4	1	2.28
PLOD1	procollagen-lysine, 2-oxoglutarate 5-dioxygenase 1	1	1	2.28
ATXN2	ataxin 2	0	1	2.1375
MTA2	metastasis-associated gene family, member 2	0	1	2.1375
NRGN	neurogranin	0	1	2.1375
BRPF1	bromodomain and PHD finger containing, 1	0	1	2.09388
RGS17	regulator of G-protein signaling 17	0	1	2.09388
PTK2B	PTK2 protein tyrosine kinase 2 beta	5	1	2.052
SPATS2	spermatogenesis associated, serine-rich 2	0	1	2.052
CACHD1	cache domain containing 1	0	1	1.93585
TGIF1	TGFB-induced factor homeobox 1	0	1	1.9
STRN3	striatin, calmodulin binding protein 3	0	1	1.86546
SLC35A4	solute carrier family 35, member A4	0	1	1.83214
CPSF4	cleavage and polyadenylation specific factor 4	0	1	1.8
MARCKSL1	MARCKS-like 1	2	1	1.8
TFPI2	tissue factor pathway inhibitor 2	0	1	1.8
FSCN1	fascin homolog 1, actin bundling protein (Strongylocentrotus purpuratus)	0	1	1.76897
LMNB1	lamin B1	0	1	1.76897

GPX7	glutathione peroxidase 7	2	1	1.73898
OSGIN2	oxidative stress induced growth inhibitor family member 2	0	1	1.73898
FBXO9	f-box protein 9	0	1	1.71
MAF1	MAF1 homolog (S. cerevisiae)	0	1	1.71
CCNL1	cyclin L1	0	1	1.65483
CTNNBIP1	catenin beta interacting protein 1	1	2	1.6416
EGLN2	EGL nine homolog 2 (C. elegans)	2	1	1.60313
PLCL2	phospholipase C-like 2	0	1	1.60313
GPR64	G protein-coupled receptor 64	0	2	1.60312
ALDH1A2	aldehyde dehydrogenase family 1, subfamily A2	2	1	1.57846
DHX15	DEAH (Asp-Glu-Ala-His) box polypeptide 15	1	1	1.57846
HOXD10	homeobox D10	0	1	1.55455
RGS7	regulator of G protein signaling 7	0	1	1.55455
UBA6	ubiquitin-like modifier activating enzyme 6	1	2	1.54286
FXR2	fragile X mental retardation, autosomal homolog 2	0	1	1.50882
HCN3	hyperpolarization-activated, cyclic nucleotide-gated K+ 3	0	1	1.50882
NCOR2	nuclear receptor co-repressor 2	1	1	1.50882
NDFIP2	Nedd4 family interacting protein 2	0	1	1.48696
NONO	non-POU-domain-containing, octamer binding protein	0	1	1.48696
CDC42BPB	CDC42 binding protein kinase beta	0	1	1.46572
GABARAPL2	gamma-aminobutyric acid (GABA) A receptor-associated protein-like 2	1	1	1.46572
GPBP1	GC-rich promoter binding protein 1	0	1	1.46572
SMAP1	stromal membrane-associated protein 1	1	1	1.425
LDHA	lactate dehydrogenase A	5	1	1.40548
CUL3	cullin 3	1	1	1.38648
CAR2	carbonic anhydrase 2	4	1	1.368
TRIM63	tripartite motif-containing 63	0	1	1.368
MDFIC	MyoD family inhibitor domain containing	0	2	1.35894
LEF1	lymphoid enhancer binding factor 1	11	1	1.35
NCOA1	nuclear receptor coactivator 1	0	1	1.35
HOXA1	homeobox A1	0	2	1.34117
ACCN1	amiloride-sensitive cation channel 1, neuronal (degenerin)	1	1	1.33247
NUMBL	numb-like	1	1	1.33247
SC4MOL	sterol-C4-methyl oxidase-like	2	1	1.31538
TMEM178	transmembrane protein 178	0	1	1.31538
BET1	blocked early in transport 1 homolog (S. cerevisiae)	1	1	1.29874
PNOC	prepronociceptin	0	1	1.29874
FAM135A	family with sequence similarity 135, member A	0	1	1.2825

WNT1	wingless-related MMTV integration site 1	5	1	1.2825
CD163	CD163 antigen	0	1	1.26666
PTGER4	prostaglandin E receptor 4 (subtype EP4)	1	1	1.26666
SLC6A14	solute carrier family 6 (neurotransmitter transporter), member 14	0	1	1.26666
ARSJ	arylsulfatase J	0	1	1.25122
B4GALT2	UDP-Gal:betaGlcNAc beta 1,4- galactosyltransferase, polypeptide 2	5	1	1.25122
RALGDS	ral guanine nucleotide dissociation stimulator	3	1	1.25122
YTHDF2	YTH domain family 2	0	1	1.25122
CALCR	calcitonin receptor	1	2	1.24364
GIT1	G protein-coupled receptor kinase-interactor 1	2	1	1.20706
NPEPPS	aminopeptidase puromycin sensitive	0	1	1.20706
PDCD4	programmed cell death 4	0	1	1.20706
SNX5	sorting nexin 5	0	1	1.20706
KDM6A	4lysine (K)-specific demethylase 6A	0	1	1.17931
AMBRA1	autophagy/beclin 1 regulator 1	0	1	1.16591
CFL2	cofilin 2, muscle	3	2	1.15932
E2F5	E2F transcription factor 5	2	1	1.15281
CAPRIN1	cell cycle associated protein 1	0	2	1.14637
RCN1	reticulocalbin 1	0	1	1.14
TUSC2	tumor suppressor candidate 2	0	1	1.12747
WASF1	WASP family 1	4	1	1.12747
DBC1	deleted in bladder cancer 1 (human)	0	1	1.11522
FAM108C	family with sequence similarity 108, member C	0	1	1.11522
SLITRK4	SLIT and NTRK-like family, member 4	0	1	1.11522
USP46	ubiquitin specific peptidase 46	0	2	1.11522
EI24	etoposide induced 2.4 mRNA	1	1	1.10322
GPR85	G protein-coupled receptor 85	0	1	1.10322
HNF4G	hepatocyte nuclear factor 4, gamma	1	1	1.10322
IDH1	isocitrate dehydrogenase 1 (NADP+), soluble	4	1	1.10322
CACNB3	calcium channel, voltage-dependent, beta 3 subunit	5	1	1.09149
FUT8	fucosyltransferase 8	3	1	1.09149
ELF5	E74-like factor 5	0	1	1.08
MEST	mesoderm specific transcript	0	1	1.08
PDIA6	protein disulfide isomerase associated 6	1	1	1.08
SRPR	signal recognition particle receptor ('docking protein')	1	1	1.08
FBN1	fibrillin 1	0	1	1.06875
GTF2H1	general transcription factor II H, polypeptide 1	2	1	1.06875
TOX3	TOX high mobility group box family member 3	0	1	1.06875

ASF1A	ASF1 anti-silencing function 1 homolog A (S. cerevisiae)	0	1	1.05773
CD320	CD320 antigen	0	1	1.05773
FAM108B	family with sequence similarity 108, member B	0	1	1.05773
RNF43	ring finger protein 43	0	1	1.05773
UBE2D1	ubiquitin-conjugating enzyme E2D 1, UBC4/5 homolog (yeast)	2	1	1.05773
NUAK1	NUAK family, SNF1-like kinase, 1	0	2	1.05231
ELF2	E74-like factor 2	0	1	1.04694
HLTF	helicase-like transcription factor	0	1	1.04694
BCKDK	branched chain ketoacid dehydrogenase kinase	0	1	1.03637
ABR	active BCR-related gene	0	2	1.026
PAQR9	progestin and adipoQ receptor family member IX	0	1	1.026
ZCCHC17	zinc finger, CCHC domain containing 17	0	1	1.026
DACH1	dachshund 1 (Drosophila)	0	2	1.0209
ANGPT2	angiopoietin 2	0	1	1.01584
GGNBP2	gametogenetin binding protein 2	0	1	1.01584
KLF3	Kruppel-like factor 3 (basic)	0	1	1.01584
TAF5	TAF5 RNA polymerase II, TATA box binding protein (TBP)-associated factor	1	1	1.01584
G3BP2	GTPase activating protein (SH3 domain) binding protein 2	0	2	1.01084
FUNDC2	FUN14 domain containing 2	0	1	1.00588
PPP1R10	protein phosphatase 1, regulatory subunit 10	0	1	1.00588
REV3L	REV3-like, catalytic subunit of DNA polymerase zeta RAD54 like (S. cerevisiae)	1	1	1.00588
SLITRK6	SLIT and NTRK-like family, member 6	0	1	1.00588
TNFRSF11B	tumor necrosis factor receptor superfamily, member 11b (osteoprotegerin)	1	1	1.00588
EPB4.1L5	erythrocyte protein band 4.1-like 5	0	1	0.996115
KCNK5	potassium channel, subfamily K, member 5	1	1	0.986539
MET	met proto-oncogene	10	2	0.981819
KCNK3	potassium channel, subfamily K, member 3	0	1	0.977145
SGSM2	small G protein signaling modulator 2	0	1	0.977145
FLI1	Friend leukemia integration 1	0	1	0.958879
CNTNAP1	contactin associated protein-like 1	1	1	0.950001
GALNT1	UDP-N-acetyl-alpha-D-galactosamine:polypeptide N-acetylgalactosaminyltransferase 1	2	1	0.950001
PICALM	phosphatidylinositol binding clathrin assembly protein	0	1	0.950001
HS6ST2	heparan sulfate 6-O-sulfotransferase 2	1	2	0.949999
NDST1	N-deacetylase/N-sulfotransferase (heparan glucosaminyl) 1	2	2	0.945622
CCDC97	coiled-coil domain containing 97	0	1	0.941283
IRF2	interferon regulatory factor 2	0	1	0.941283
SDHC	succinate dehydrogenase complex, subunit C, integral membrane protein	6	1	0.932727

TOX	thymocyte selection-associated high mobility group box	0	1	0.932727
ACSL4	acyl-CoA synthetase long-chain family member 4	5	2	0.928507
ATP6V1B2	ATPase, H+ transporting, lysosomal V1 subunit B2	4	1	0.924325
INPP5A	inositol polyphosphate-5-phosphatase A	3	1	0.924325
PLAGL1	pleiomorphic adenoma gene-like 1	0	1	0.924325
TCFAP2C	transcription factor AP-2, gamma	0	1	0.924325
HTR2C	5-hydroxytryptamine (serotonin) receptor 2C	3	2	0.924323
ARHGAP1	Rho GTPase activating protein 1	0	1	0.91607
MAP2K1	mitogen-activated protein kinase kinase 1	37	1	0.91607
P4HA1	procollagen-proline, 2-oxoglutarate 4-dioxygenase (proline 4-hydroxylase), alpha 1 polypeptide	2	1	0.91607
SEMA4C	sema domain, immunoglobulin domain (Ig), transmembrane domain (TM) and short cytoplasmic domain, (semaphorin) 4C	1	1	0.91607
AXL	AXL receptor tyrosine kinase	0	1	0.907964
CNNM4	cyclin M4	0	1	0.907964
DUSP6	dual specificity phosphatase 6	1	1	0.907964
LBR	lamin B receptor	0	1	0.907964
OCLN	occludin	4	1	0.900001
NAA50	N(alpha)-acetyltransferase 50, NatE catalytic subunit	0	2	0.896069
CCNL2	cyclin L2	0	1	0.892172
DLX1	distal-less homeobox 1	0	1	0.892172
SPOP	speckle-type POZ protein	0	1	0.892172
RNF216	ring finger protein 216	0	1	0.884482
SLC27A4	solute carrier family 27 (fatty acid transporter), member 4	1	1	0.884482
ANKRD13C	ankyrin repeat domain 13c	0	1	0.876924
CTCF	CCCTC-binding factor	0	1	0.876924
ACTR1A	ARP1 actin-related protein 1 homolog A, centractin alpha (yeast)	0	1	0.862184
NAA15	N(alpha)-acetyltransferase 15, NatA auxiliary subunit	0	1	0.862184
RBPMS2	RNA binding protein with multiple splicing 2	0	1	0.855
TM9SF4	transmembrane 9 superfamily protein member 4	0	1	0.847935
CCNE2	cyclin E2	6	1	0.840983
CHST10	carbohydrate sulfotransferase 10	0	1	0.840983
PKIA	protein kinase inhibitor, alpha	0	2	0.840983
ACSL1	acyl-CoA synthetase long-chain family member 1	5	1	0.834147
EIF2C4	eukaryotic translation initiation factor 2C, 4	0	1	0.834147
FIGN	fidgetin	0	1	0.834147
INO80	INO80 homolog (S. cerevisiae)	0	1	0.834147
SERPINE1	serine (or cysteine) peptidase inhibitor, clade E, member 1	3	1	0.834147

SOX9	SRY-box containing gene 9	0	1	0.834147
B3GALT2	UDP-Gal:betaGlcNAc beta 1,3-galactosyltransferase, polypeptide 2	2	1	0.827421
UBP1	upstream binding protein 1	0	1	0.827421
KLF11	Kruppel-like factor 11	0	1	0.820799
SETD8	SET domain containing (lysine methyltransferase) 8	1	1	0.820799
TMOD1	tropomodulin 1	0	1	0.820799
LRIG1	leucine-rich repeats and immunoglobulin-like domains 1	0	1	0.814286
POU4F2	POU domain, class 4, transcription factor 2	0	1	0.814286
SENP2	SUMO/sentrin specific peptidase 2	2	1	0.807875
CYTH1	cytohesin 1	0	1	0.795349
EPS15	epidermal growth factor receptor pathway substrate 15	1	1	0.795349
LARP4B	La ribonucleoprotein domain family, member 4B	0	1	0.795349
RRAS2	related RAS viral (r-ras) oncogene homolog 2	3	1	0.795349
BCL6	B-cell leukemia/lymphoma 6	0	1	0.789232
CS	citrate synthase	3	1	0.789232
ELMOD1	ELMO domain containing 1	0	1	0.789232
NOTCH1	Notch gene homolog 1 (Drosophila)	3	1	0.789232
CSNK2A2	casein kinase 2, alpha prime polypeptide	3	1	0.783205
EBF2	early B-cell factor 2	0	1	0.783205
TMPO	thymopoietin	0	1	0.783205
GRM7	glutamate receptor, metabotropic 7	1	1	0.777273
MCFD2	multiple coagulation factor deficiency 2	0	1	0.777273
MEIS2	Meis homeobox 2	0	1	0.777273
NACC1	nucleus accumbens associated 1, BEN and BTB (POZ) domain containing	0	1	0.777273
PPP1CB	protein phosphatase 1, catalytic subunit, beta isoform	6	1	0.777273
TJP1	tight junction protein 1	3	1	0.777273
JHDM1D	jumonji C domain-containing histone demethylase 1 homolog D (S. cerevisiae)	0	1	0.771429
MYO1C	myosin IC	0	1	0.76567
TBR1	T-box brain gene 1	0	1	0.76
TGFBR2	transforming growth factor, beta receptor II	10	2	0.757196
CHUK	conserved helix-loop-helix ubiquitous kinase	19	1	0.754412
DAB2	disabled homolog 2 (Drosophila)	1	1	0.754412
TMEM50B	transmembrane protein 50B	0	1	0.754412
VAMP2	vesicle-associated membrane protein 2	3	1	0.754412
LGR4	leucine-rich repeat-containing G protein-coupled receptor 4	1	1	0.748904
FAM175B	family with sequence similarity 175, member B	0	1	0.743478
LENG8	leukocyte receptor cluster (LRC) member 8	0	1	0.743478
LPHN2	latrophilin 2	0	1	0.743478

TSHZ3	teashirt zinc finger family member 3	0	1	0.743478
STX12	syntaxin 12	1	1	0.73813
TMEM55A	transmembrane protein 55A	0	1	0.73813
ADRA2B	adrenergic receptor, alpha 2b	1	1	0.732858
GJA1	gap junction protein, alpha 1	2	1	0.732858
TLK1	tousled-like kinase 1	0	1	0.732858
JAG1	jagged 1	1	1	0.727659
PROSAPIP1	ProSAPiP1 protein	0	1	0.727659
SDC1	syndecan 1	3	1	0.727659
GATA6	GATA binding protein 6	0	1	0.722536
RIC8B	resistance to inhibitors of cholinesterase 8 homolog B (C. elegans)	0	1	0.722536
SGK1	serum/glucocorticoid regulated kinase 1	1	1	0.722536
ZDHHC17	zinc finger, DHHC domain containing 17	0	1	0.722536
ANKRD28	ankyrin repeat domain 28	0	1	0.717484
DNAJC6	DnaJ (Hsp40) homolog, subfamily C, member 6	1	1	0.717484
MAP3K3	mitogen-activated protein kinase kinase kinase 3	3	1	0.717484
METAP1	methionyl aminopeptidase 1	0	1	0.717484
MYRIP	myosin VIIA and Rab interacting protein	0	1	0.717484
JAZF1	JAZF zinc finger 1	0	1	0.712499
PROSC	proline synthetase co-transcribed	0	1	0.712499
SLC25A36	solute carrier family 25, member 36	0	1	0.712499
ACBD3	acyl-Coenzyme A binding domain containing 3	0	1	0.702741
DPYSL2	dihydropyrimidinase-like 2	1	1	0.702741
MAT2A	methionine adenosyltransferase II, alpha	3	1	0.702741
FOSB	FBJ osteosarcoma oncogene B	0	1	0.697958
TEAD1	TEA domain family member 1	0	1	0.697958
SOX11	SRY-box containing gene 11	0	2	0.695593
TRIM2	tripartite motif-containing 2	0	2	0.68859
ADD3	adducin 3 (gamma)	0	1	0.683999
RAD21	RAD21 homolog (S. pombe)	1	1	0.683999
VCL	vinculin	6	1	0.683999
CNTNAP2	contactin associated protein-like 2	1	1	0.67947
JARID2	jumonji, AT rich interactive domain 2	0	1	0.67947
MAP3K12	mitogen-activated protein kinase kinase kinase 12	1	1	0.675001
TIAM1	T-cell lymphoma invasion and metastasis 1	2	1	0.675001
CCNG1	cyclin G1	1	1	0.670587
GALNT7	UDP-N-acetyl-alpha-D-galactosamine: polypeptide N-acetylgalactosaminyltransferase 7	2	1	0.670587

RRAGC	Ras-related GTP binding C	0	1	0.670587
FKBP5	FK506 binding protein 5	0	1	0.666234
RAI14	retinoic acid induced 14	0	1	0.666234
GOLPH3L	golgi phosphoprotein 3-like	0	1	0.661936
NEK6	NIMA (never in mitosis gene a)-related expressed kinase 6	0	1	0.661936
SMARCC1	SWI/SNF related, matrix associated, actin dependent regulator of chromatin, subfamily c, member 1	0	1	0.661936
PPP2R3A	protein phosphatase 2, regulatory subunit B'', alpha	0	1	0.657691
SEMA4F	sema domain, immunoglobulin domain (Ig), TM domain, and short cytoplasmic domain	1	1	0.657691
BTBD11	BTB (POZ) domain containing 11	0	1	0.653503
DIP2C	DIP2 disco-interacting protein 2 homolog C (Drosophila)	0	1	0.653503
CITED2	Cbp/p300-interacting transactivator, with Glu/Asp-rich carboxy-terminal domain, 2	0	1	0.649368
ZMYND11	zinc finger, MYND domain containing 11	0	1	0.649368
MAPRE1	microtubule-associated protein, RP/EB family, member 1	0	1	0.645282
SLC1A1	solute carrier family 1 (neuronal/epithelial high affinity glutamate transporter, system Xag), member 1	1	1	0.645282
DMBX1	diencephalon/mesencephalon homeobox 1	0	1	0.637267
SAR1A	SAR1 gene homolog A (S. cerevisiae)	1	1	0.633334
UBAP2	ubiquitin-associated protein 2	0	1	0.633334
SLMAP	sarcolemma associated protein	0	1	0.629447
GRB10	growth factor receptor bound protein 10	0	1	0.62561
TNFAIP3	tumor necrosis factor, alpha-induced protein 3	1	1	0.62561
BRPF3	bromodomain and PHD finger containing, 3	0	1	0.621819
SMAD3	MAD homolog 3 (Drosophila)	10	1	0.621819
YES1	Yamaguchi sarcoma viral (v-yes) oncogene homolog 1	2	1	0.621819
LANCL1	LanC (bacterial lantibiotic synthetase component C)-like 1	0	1	0.610715
RAB43	RAB43, member RAS oncogene family	0	1	0.603529
FZD7	frizzled homolog 7 (Drosophila)	4	1	0.600001
IVNS1ABP	influenza virus NS1A binding protein	0	1	0.600001
MPP2	membrane protein, palmitoylated 2 (MAGUK p55 subfamily member 2)	0	1	0.600001
PAK2	p21 protein (Cdc42/Rac)-activated kinase 2	7	1	0.600001
SIRPA	signal-regulatory protein alpha	0	1	0.600001
ZEB1	zinc finger E-box binding homeobox 1	0	1	0.600001
FAM70A	family with sequence similarity 70, member A	0	1	0.596511
FNDC3A	fibronectin type III domain containing 3A	0	1	0.596511
PPP1R16B	protein phosphatase 1, regulatory (inhibitor) subunit 16B	0	1	0.596511
CPLX2	complexin 2	0	1	0.593064

MEIS1	Meis homeobox 1	0	1	0.593064
RUNX1T1	runt-related transcription factor 1; translocated to, 1 (cyclin D-related)	2	1	0.593064
CCND1	cyclin D1	19	1	0.586285
ERBB2IP	Erbb2 interacting protein	1	1	0.586285
PRDM1	PR domain containing 1, with ZNF domain	0	1	0.586285
PRPF4B	PRP4 pre-mRNA processing factor 4 homolog B (yeast)	0	1	0.586285
KPNA4	karyopherin (importin) alpha 4	0	1	0.582954
LASS6	LAG1 homolog, ceramide synthase 6	0	1	0.579661
TOP1	topoisomerase (DNA) I	0	1	0.579661
LDLRAD3	low density lipoprotein receptor class A domain containing 3	0	1	0.576404
YWHAG	tyrosine 3-monooxygenase/tryptophan 5-monooxygenase activation protein, gamma polypeptide	3	1	0.576404
NFAT5	nuclear factor of activated T-cells 5	6	2	0.57479
CAMK2G	calcium/calmodulin-dependent protein kinase II gamma	11	1	0.56685
CLDN12	claudin 12	0	1	0.563736
MYH9	myosin, heavy polypeptide 9, non-muscle	3	1	0.563736
PEA15A	phosphoprotein enriched in astrocytes 15A	0	1	0.563736
RAD23B	RAD23b homolog (S. cerevisiae)	2	1	0.563736
CLCN3	chloride channel 3	0	1	0.560656
PIP4K2B	phosphatidylinositol-5-phosphate 4-kinase, type II, beta	3	1	0.560656
ERLIN2	ER lipid raft associated 2	0	1	0.557609
SPOCK1	sparc/osteonectin, cwcv and kazal-like domains proteoglycan 1	0	1	0.557609
FOXJ2	forkhead box J2	0	1	0.554594
MARCKS	myristoylated alanine rich protein kinase C substrate	1	1	0.554594
ELF4	E74-like factor 4 (ets domain transcription factor)	0	1	0.548664
ARHGAP20	Rho GTPase activating protein 20	0	1	0.545744
GNG2	guanine nucleotide binding protein (G protein), gamma 2	1	1	0.545744
SOCS6	suppressor of cytokine signaling 6	0	1	0.545744
CTNND1	catenin (cadherin associated protein), delta 1	2	1	0.542857
ITCH	itchy, E3 ubiquitin protein ligase	2	1	0.542857
NRIP3	nuclear receptor interacting protein 3	0	1	0.542857
BDNF	brain derived neurotrophic factor	3	1	0.540001
HAS2	hyaluronan synthase 2	0	1	0.540001
PAX9	paired box gene 9	0	1	0.534375
SEC24A	Sec24 related gene family, member A (S. cerevisiae)	1	1	0.534375
BNIP3L	BCL2/adenovirus E1B interacting protein 3-like	0	1	0.528865
TMEM64	transmembrane protein 64	0	1	0.528865
MAP3K7	mitogen-activated protein kinase kinase kinase 7	9	1	0.526154

ARHGAP24	Rho GTPase activating protein 24	0	1	0.52347
CTDSPL	CTD (carboxy-terminal domain, RNA polymerase II, polypeptide A) small phosphatase-like	0	1	0.52347
NAV1	neuron navigator 1	0	1	0.52347
VAT1	vesicle amine transport protein 1 homolog (T californica)	0	1	0.52347
CLIC4	chloride intracellular channel 4 (mitochondrial)	0	1	0.520812
PDE4B	phosphodiesterase 4B, cAMP specific	1	1	0.520812
SLC39A10	solute carrier family 39 (zinc transporter), member 10	0	1	0.520812
ST18	suppression of tumorigenicity 18	0	1	0.518182
ZFP367	zinc finger protein 367	0	1	0.518182
MED1	mediator complex subunit 1	0	1	0.515578
ACVR1B	activin A receptor, type 1B	1	1	0.507921
ETV1	ets variant gene 1	0	1	0.507921
TNRC6B	trinucleotide repeat containing 6b	0	2	0.505419
INTU	inturned planar cell polarity effector homolog (Drosophila)	0	1	0.505418
CNTN2	contactin 2	1	1	0.502941
ERLIN1	ER lipid raft associated 1	0	1	0.502941
PIK3R3	phosphatidylinositol 3 kinase, regulatory subunit, polypeptide 3 (p55)	39	1	0.502941
BAZ2A	bromodomain adjacent to zinc finger domain, 2A	0	1	0.500488
DAAM1	dishevelled associated activator of morphogenesis 1	1	1	0.500488
SRPK2	serine/arginine-rich protein specific kinase 2	0	1	0.500488
TRIB1	tribbles homolog 1 (Drosophila)	0	1	0.500488
GREM2	gremlin 2 homolog, cysteine knot superfamily (Xenopus laevis)	0	1	0.498059
PAK3	p21 protein (Cdc42/Rac)-activated kinase 3	6	1	0.498059
GPR158	G protein-coupled receptor 158	0	1	0.495652
KPNA1	karyopherin (importin) alpha 1	0	1	0.495652
CREBZF	CREB/ATF bZIP transcription factor	0	1	0.49091
DDX17	DEAD (Asp-Glu-Ala-Asp) box polypeptide 17	0	1	0.488571
GPRC5B	G protein-coupled receptor, family C, group 5, member B	0	1	0.488571
MMGT1	membrane magnesium transporter 1	0	1	0.488571
SYS1	SYS1 Golgi-localized integral membrane protein homolog (S. cerevisiae)	0	1	0.486256
SEMA6A	sema domain, transmembrane domain (TM), and cytoplasmic domain, (semaphorin) 6A	1	1	0.483963
MAP3K1	mitogen-activated protein kinase kinase kinase 1	5	1	0.48169
FMR1	fragile X mental retardation syndrome 1 homolog	0	1	0.479439
KLHL18	kelch-like 18 (Drosophila)	0	1	0.479439
ACVR2A	activin receptor IIA	2	1	0.475
AKAP6	A kinase (PRKA) anchor protein 6	0	1	0.475

PTGFR	prostaglandin F receptor	2	1	0.475
TGFBR3	transforming growth factor, beta receptor III	0	1	0.475
ARHGAP26	Rho GTPase activating protein 26	0	1	0.472811
STK4	serine/threonine kinase 4	3	1	0.472811
FOXO1	forkhead box O1	3	1	0.464254
PPP2R5E	protein phosphatase 2, regulatory subunit B (B56), epsilon isoform	2	1	0.464254
FBXO41	F-box protein 41	0	1	0.462162
ELAVL2	ELAV (embryonic lethal, abnormal vision, Drosophila)-like 2 (Hu antigen B)	0	1	0.46009
FOXK1	forkhead box K1	0	1	0.46009
KITL	kit ligand	4	1	0.458036
MBTD1	mbt domain containing 1	0	1	0.458036
PDGFRA	platelet derived growth factor receptor, alpha polypeptide	11	1	0.458036
CGGBP1	CGG triplet repeat binding protein 1	0	1	0.456001
MBNL1	muscleblind-like 1 (Drosophila)	0	1	0.456001
CADM1	cell adhesion molecule 1	1	1	0.453982
BAZ2B	bromodomain adjacent to zinc finger domain, 2B	0	1	0.451982
LAMC1	laminin, gamma 1	7	1	0.451982
ZFHX4	zinc finger homeodomain 4	0	1	0.45
FNBP1L	formin binding protein 1-like	0	1	0.448035
KBTBD8	kelch repeat and BTB (POZ) domain containing 8	0	1	0.448035
SCN2B	sodium channel, voltage-gated, type II, beta	0	1	0.448035
NDRG3	N-myc downstream regulated gene 3	0	1	0.446087
SYT1	synaptotagmin I	0	1	0.446087
PHF15	PHD finger protein 15	0	1	0.442241
RAB14	RAB14, member RAS oncogene family	0	1	0.442241
ZFP238	zinc finger protein 238	0	1	0.442241
GMFB	glia maturation factor, beta	0	1	0.440343
TMCC3	transmembrane and coiled coil domains 3	0	1	0.440343
TNPO1	transportin 1	0	1	0.438462
HIC2	hypermethylated in cancer 2	0	1	0.434746
ACAP2	ArfGAP with coiled-coil, ankyrin repeat and PH domains 2	1	1	0.432912
OSBPL8	oxysterol binding protein-like 8	0	1	0.432912
SRSF1	serine/arginine-rich splicing factor 1	1	1	0.432912
ANKFY1	ankyrin repeat and FYVE domain containing 1	0	1	0.431092
NACC2	nucleus accumbens associated 2, BEN and BTB (POZ) domain containing	0	1	0.4275
RBM25	RNA binding motif protein 25	1	1	0.4275
ZFHX3	zinc finger homeobox 3	0	1	0.425726
MAF	avian musculoaponeurotic fibrosarcoma (v-maf) AS42 oncogene homolog	0	1	0.423967

ASB1	ankyrin repeat and SOCS box-containing 1	0	1	0.420491
RAP2A	RAS related protein 2a	0	1	0.418775
FOXP2	forkhead box P2	0	1	0.417073
DCAF7	DDB1 and CUL4 associated factor 7	0	1	0.415385
RYBP	RING1 and YY1 binding protein	0	1	0.413709
ESRRG	estrogen-related receptor gamma	0	1	0.408765
BCL2L11	BCL2-like 11 (apoptosis facilitator)	0	1	0.400782
FNDC3B	fibronectin type III domain containing 3B	0	1	0.400782
NEGR1	neuronal growth regulator 1	1	1	0.399222
STK35	serine/threonine kinase 35	0	1	0.396139
TAB3	TGF-beta activated kinase 1/MAP3K7 binding protein 3	1	1	0.391603
CAMK1D	calcium/calmodulin-dependent protein kinase ID	0	1	0.38717
FBXO28	F-box protein 28	0	1	0.385714
ROBO2	roundabout homolog 2 (Drosophila)	1	1	0.385714
FAM126A	family with sequence similarity 126, member A	0	1	0.38427
MYO5A	myosin VA	0	1	0.38427
CACNA1E	calcium channel, voltage-dependent, R type, alpha 1E subunit	3	1	0.375824
PLAGL2	pleiomorphic adenoma gene-like 2	0	1	0.369065
MLLT3	myeloid/lymphoid or mixed-lineage leukemia (trithorax homolog, Drosophila); translocated to, 3	0	1	0.361268
WNK1	WNK lysine deficient protein kinase 1	0	1	0.36
NR6A1	nuclear receptor subfamily 6, group A, member 1	0	1	0.357491
CXCL12	chemokine (C-X-C motif) ligand 12	5	1	0.355017
CLOCK	circadian locomotor output cycles kaput	1	1	0.344295
DNM3	dynamin 3	3	1	0.340864
MTF1	metal response element binding transcription factor 1	0	1	0.339735
CAMTA1	calmodulin binding transcription activator 1	0	1	0.3375
FRMD4A	FERM domain containing 4A	0	1	0.3375
GABRB2	gamma-aminobutyric acid (GABA) A receptor, subunit beta 2	1	1	0.327796
CSNK1G1	casein kinase 1, gamma 1	1	1	0.323659
QK	quaking	0	1	0.319626
PTEN	phosphatase and tensin homolog	11	1	0.30997
FUT9	fucosyltransferase 9	3	1	0.301765
CELF2	CUGBP, Elav-like family member 2	0	1	0.298256
DCX	doublecortin	0	1	0.295677
TBL1XR1	transducin (beta)-like 1X-linked receptor 1	1	1	0.294827
ITSN1	intersectin 1 (SH3 domain protein 1A)	0	1	0.278049

Supplemental Table S3. Significantly enriched pathways that regulate these targets in the functional annotation.

Supplemental Table S3A. C57BL6.

CATEGORY	TERM	TERM_ID	URL	TOTAL_GENES_OF_THE_TERM	UNION_TARGETS_IN_THE_TERM	MIRS_IN_THE_TERM	SCORE
KEGG	T_CELL_RECEPTOR_SIGNALING_PATHWAY	4660	http://www.kegg.jp/kegg-bin/show_pathway?mmu04660+mmu:12402+mmu:12540+mmu:17974+mmu:18481+mmu:26409+mmu:26417+mmu:54446	109	7	3	1.869
KEGG	AXON_GUIDANCE	4360	http://www.kegg.jp/kegg-bin/show_pathway?mmu04360+mmu:12540+mmu:12632+mmu:12934+mmu:17295+mmu:17974+mmu:18481+mmu:20358+mmu:214968+mmu:26417+mmu:54446	131	10	4	1.671
KEGG	FOCAL_ADHESION	4510	http://www.kegg.jp/kegg-bin/show_pathway?mmu04510+mmu:107589+mmu:11461+mmu:12540+mmu:16000+mmu:17295+mmu:18481+mmu:18590+mmu:21825+mmu:22339+mmu:226519+mmu:26417	197	11	3	1.436
KEGG	CELL_ADHESION_MOLECULES_(CAMS)	4514	http://www.kegg.jp/kegg-bin/show_pathway?mmu04514+mmu:17528+mmu:18260+mmu:20969+mmu:320840	149	4	3	1.428
KEGG	ENDOCYTOSIS	4144	http://www.kegg.jp/kegg-bin/show_pathway?mmu04144+mmu:12402+mmu:12540+mmu:13132+mmu:15511+mmu:17127+mmu:17295+mmu:216963+mmu:21813+mmu:26385+mmu:67300+mmu:78618+mmu:98366	219	12	4	1.416
KEGG	REGULATION_OF_ACTIN_CYTOSKELETON	4810	http://www.kegg.jp/kegg-bin/show_pathway?mmu04810+mmu:107589+mmu:11461+mmu:12540+mmu:12632+mmu:14169+mmu:18481+mmu:18590+mmu:19241+mmu:216963+mmu:21844+mmu:26417	215	11	4	1.379
KEGG	RENAL_CELL_CARCINOMA	5211	http://www.kegg.jp/kegg-bin/show_pathway?mmu05211+mmu:12540+mmu:17295+mmu:18481+mmu:18590+mmu:22339+mmu:23871+mmu:26417	71	7	2	1.133
KEGG	WNT_SIGNALING_PATHWAY	4310	http://www.kegg.jp/kegg-bin/show_pathway?mmu04310+mmu:12325+mmu:14	153	9	4	1.065

			369+mmu:17127+mmu:20377+mmu:208846+mmu:26409+mmu:54446+mmu:67087+mmu:75826				
KEGG	VEGF_SIGNALING_PATHWAY	4370	http://www.kegg.jp/kegg-bin/show_pathway?mmu04370+mmu:12540+mmu:22339+mmu:26417+mmu:54446	76	4	3	0.998
KEGG	ERBB_SIGNALING_PATHWAY	4012	http://www.kegg.jp/kegg-bin/show_pathway?mmu04012+mmu:12325+mmu:12402+mmu:17974+mmu:18481+mmu:26417	87	5	3	0.941
KEGG	B_CELL_RECEPTOR_SIGNALING_PATHWAY	4662	http://www.kegg.jp/kegg-bin/show_pathway?mmu04662+mmu:26417+mmu:54446	76	2	3	0.908
KEGG	SALIVARY_SECRETION	4970	http://www.kegg.jp/kegg-bin/show_pathway?mmu04970+mmu:12313+mmu:12314+mmu:22318+mmu:64177	77	4	2	0.894
KEGG	ADIPOCYTOKINE_SIGNALING_PATHWAY	4920	http://www.kegg.jp/kegg-bin/show_pathway?mmu04920+mmu:12675+mmu:14081+mmu:50790	68	4	3	0.889
KEGG	NON-SMALL_CELL_LUNG_CANCER	5223	http://www.kegg.jp/kegg-bin/show_pathway?mmu05223+mmu:12443+mmu:18710+mmu:26395+mmu:58231	54	4	3	0.883
KEGG	ECM-RECEPTOR_INTERACTION	4512	http://www.kegg.jp/kegg-bin/show_pathway?mmu04512+mmu:20969+mmu:21825+mmu:226519	83	3	3	0.876
KEGG	GLYCOLYSIS_GLUCONEOGENESIS	10	http://www.kegg.jp/kegg-bin/show_pathway?mmu00010+mmu:11674	60	1	1	0.849
KEGG	TOXOPLASMOSIS	5145	http://www.kegg.jp/kegg-bin/show_pathway?mmu05145+mmu:15511+mmu:226519+mmu:26409+mmu:26417	128	4	3	0.752
KEGG	SMALL_CELL_LUNG_CANCER	5222	http://www.kegg.jp/kegg-bin/show_pathway?mmu05222+mmu:226519	85	1	1	0.74
KEGG	MAPK_SIGNALING_PATHWAY	4010	http://www.kegg.jp/kegg-bin/show_pathway?mmu04010+mmu:12064+mmu:12540+mmu:14169+mmu:15511+mmu:16478+mmu:18590+mmu:21813+mmu:26404+mmu:26406+mmu:26409+mmu:26417+mmu:67603	271	12	4	0.738

KEGG	ARACHIDONIC_ACID_METABOLISM	590	http://www.kegg.jp/kegg-bin/show_pathway?mmu00590+mmu:67305	86	1	1	0.736
KEGG	PATHWAYS_IN_CANCER	5200	http://www.kegg.jp/kegg-bin/show_pathway?mmu05200+mmu:12402+mmu:12540+mmu:14169+mmu:14369+mmu:16000+mmu:17127+mmu:17295+mmu:18590+mmu:21813+mmu:22027+mmu:22339+mmu:226519+mmu:26417+mmu:56458	323	14	3	0.685
KEGG	NATURAL_KILLER_CELL_MEDIATED_CYTOTOXICITY	4650	http://www.kegg.jp/kegg-bin/show_pathway?mmu04650+mmu:26417+mmu:54446	150	2	3	0.679
KEGG	LEUKOCYTE_TRANSENDOTHELIAL_MIGRATION	4670	http://www.kegg.jp/kegg-bin/show_pathway?mmu04670+mmu:11461+mmu:12388+mmu:12540+mmu:18260	120	4	3	0.67
KEGG	TIGHT_JUNCTION	4530	http://www.kegg.jp/kegg-bin/show_pathway?mmu04530+mmu:11461+mmu:12540+mmu:18260+mmu:192195+mmu:22612	135	5	3	0.658
KEGG	CELL_CYCLE	4110	http://www.kegg.jp/kegg-bin/show_pathway?mmu04110+mmu:17127+mmu:19357+mmu:22631	125	3	3	0.657
KEGG	AMOEBIASIS	5146	http://www.kegg.jp/kegg-bin/show_pathway?mmu05146+mmu:226519	115	1	1	0.65
KEGG	HEPATITIS_C	5160	http://www.kegg.jp/kegg-bin/show_pathway?mmu05160+mmu:18260+mmu:26417	137	2	2	0.63

Supplemental Table S3B. TLR5.

CATEGORY	TERM	TERM_ID	URL	TOTAL_GENES_OF_THE_TERM	UNION_TARGETS_IN_THE_TERM	MIRS_IN_THE_TERM	SCORE
KEGG	T_CELL_RECEPTOR_SIGNALING_PATHWAY	4660	http://www.kegg.jp/kegg-bin/show_pathway?mmu04660+mmu:18481+mmu:54446	109	2	1	1.864

KEGG	AXON_GUIDANCE	4360	http://www.kegg.jp/kegg-bin/show_pathway?mmu04360+mmu:18481+mmu:54446	131	2	1	1.719
KEGG	CELL_ADHESION_MOLECULES_(CAMS)	4514	http://www.kegg.jp/kegg-bin/show_pathway?mmu04514+mmu:18260+mmu:320840	149	2	1	1.62
KEGG	FOCAL_ADHESION	4510	http://www.kegg.jp/kegg-bin/show_pathway?mmu04510+mmu:18481+mmu:226519	197	2	1	1.41
KEGG	REGULATION_OF_ACTIN_CYTOSKELETON	4810	http://www.kegg.jp/kegg-bin/show_pathway?mmu04810+mmu:18481+mmu:216963	215	2	1	1.346
KEGG	ENDOCYTOSIS	4144	http://www.kegg.jp/kegg-bin/show_pathway?mmu04144+mmu:216963+mmu:78618	219	2	1	1.333
KEGG	ARGININE_AND_PROLINE_METABOLISM	330	http://www.kegg.jp/kegg-bin/show_pathway?mmu00330+mmu:18451	53	1	1	1.087
KEGG	GLUTATHIONE_METABOLISM	480	http://www.kegg.jp/kegg-bin/show_pathway?mmu00480+mmu:67305	54	1	1	1.079
KEGG	GLYCOLYSIS_GLUCONEOGENESIS	10	http://www.kegg.jp/kegg-bin/show_pathway?mmu00010+mmu:11674	60	1	1	1.038
KEGG	RENAL_CELL_CARCINOMA	5211	http://www.kegg.jp/kegg-bin/show_pathway?mmu05211+mmu:18481	71	1	1	0.973
KEGG	B_CELL_RECEPTOR_SIGNALING_PATHWAY	4662	http://www.kegg.jp/kegg-bin/show_pathway?mmu04662+mmu:54446	76	1	1	0.947
KEGG	VEGF_SIGNALING_PATHWAY	4370	http://www.kegg.jp/kegg-bin/show_pathway?mmu04370+mmu:54446	76	1	1	0.947

KEGG	SALIVARY_SECRETION	4970	http://www.kegg.jp/kegg-bin/show_pathway?mmu04970+mmu:64177	77	1	1	0.942
KEGG	ECM-RECEPTOR_INTERACTION	4512	http://www.kegg.jp/kegg-bin/show_pathway?mmu04512+mmu:226519	83	1	1	0.913
KEGG	SMALL_CELL_LUNG_CANCER	5222	http://www.kegg.jp/kegg-bin/show_pathway?mmu05222+mmu:226519	85	1	1	0.904
KEGG	ARACHIDONIC_ACID_METABOLISM	590	http://www.kegg.jp/kegg-bin/show_pathway?mmu00590+mmu:67305	86	1	1	0.9
KEGG	ERBB_SIGNALING_PATHWAY	4012	http://www.kegg.jp/kegg-bin/show_pathway?mmu04012+mmu:18481	87	1	1	0.896
KEGG	AMOEBIASIS	5146	http://www.kegg.jp/kegg-bin/show_pathway?mmu05146+mmu:226519	115	1	1	0.795
KEGG	LEUKOCYTE_TRANSENDOTHELIAL_MIGRATION	4670	http://www.kegg.jp/kegg-bin/show_pathway?mmu04670+mmu:18260	120	1	1	0.78
KEGG	CELL_CYCLE	4110	http://www.kegg.jp/kegg-bin/show_pathway?mmu04110+mmu:19357	125	1	1	0.766
KEGG	TOXOPLASMOSIS	5145	http://www.kegg.jp/kegg-bin/show_pathway?mmu05145+mmu:226519	128	1	1	0.757
KEGG	TIGHT_JUNCTION	4530	http://www.kegg.jp/kegg-bin/show_pathway?mmu04530+mmu:18260	135	1	1	0.739
KEGG	HEPATITIS_C	5160	http://www.kegg.jp/kegg-bin/show_pathway?mmu05160+mmu:18260	137	1	1	0.734
KEGG	NATURAL_KILLER_CELL_MEDIATED_CYTOTOXICITY	4650	http://www.kegg.jp/kegg-bin/show_pathway?mmu04650+mmu:54446	150	1	1	0.704

KEGG	WNT_SIGNALING_PATHWAY	4310	http://www.kegg.jp/kegg-bin/show_pathway?mmu04310+mmu:54446	153	1	1	0.698
KEGG	MAPK_SIGNALING_PATHWAY	4010	http://www.kegg.jp/kegg-bin/show_pathway?mmu04010+mmu:26404	271	1	1	0.534

Supplemental Table S3C. TLR2.

CATEGORY	TERM	TERM_ID	URL	TOTAL_GENES_OF_THE_TERM	UNION_TARGETS_IN_THE_TERM	MIRS_IN_THE_TERM	SCORE
KEGG	ARGININE_AND_PROLINE_METABOLISM	330	http://www.kegg.jp/kegg-bin/show_pathway?mmu00330+mmu:18263+mmu:18451	53	2	2	1.171
KEGG	GLUTATHIONE_METABOLISM	480	http://www.kegg.jp/kegg-bin/show_pathway?mmu00480+mmu:18263+mmu:67305	54	2	2	1.164
KEGG	T_CELL_RECEPTOR_SIGNALING_PATHWAY	4660	http://www.kegg.jp/kegg-bin/show_pathway?mmu04660+mmu:12402+mmu:12540+mmu:17974+mmu:18481+mmu:26395+mmu:26409+mmu:26417+mmu:54446	109	8	4	1.106
KEGG	AXON_GUIDANCE	4360	http://www.kegg.jp/kegg-bin/show_pathway?mmu04360+mmu:12540+mmu:17295+mmu:17974+mmu:18481+mmu:20353+mmu:20355+mmu:214968+mmu:26417+mmu:54446	131	9	4	1.098
KEGG	FOCAL_ADHESION	4510	http://www.kegg.jp/kegg-bin/show_pathway?mmu04510+mmu:107589+mmu:12540+mmu:16000+mmu:17295+mmu:	197	13	3	1.049

			18481+mmu:18590+mmu:18595+mmu:21825+mmu:22330+mmu:22339+mmu:226519+mmu:26395+mmu:26417				
KEGG	REGULATION_OF_ACTIN_CYTOSKELETON	4810	http://www.kegg.jp/kegg-bin/show_pathway?mmu04810+mmu:107589+mmu:12540+mmu:14169+mmu:17886+mmu:18481+mmu:18590+mmu:18595+mmu:19241+mmu:216963+mmu:21844+mmu:22330+mmu:26395+mmu:26417+mmu:83767	215	14	4	1.018
KEGG	ENDOCYTOSIS	4144	http://www.kegg.jp/kegg-bin/show_pathway?mmu04144+mmu:12402+mmu:12540+mmu:15511+mmu:16396+mmu:17295+mmu:18595+mmu:216963+mmu:26385+mmu:67300+mmu:78618+mmu:98366	219	11	4	0.975
KEGG	RENAL_CELL_CARCINOMA	5211	http://www.kegg.jp/kegg-bin/show_pathway?mmu05211+mmu:12540+mmu:17295+mmu:18481+mmu:18590+mmu:22339+mmu:23871+mmu:26395+mmu:26417	71	8	3	0.888
KEGG	CELL_ADHESION_MOLECULES_(CAMS)	4514	http://www.kegg.jp/kegg-bin/show_pathway?mmu04514+mmu:17528+mmu:18260+mmu:20969+mmu:21367+mmu:320840+mmu:53321+mmu:66797	149	7	4	0.875
KEGG	WNT_SIGNALING_PATHWAY	4310	http://www.kegg.jp/kegg-bin/show_pathway?mmu04310+mmu:12325+mmu:14369+mmu:16842+mmu:20377+mmu:208846+mmu:22408+mmu:26409+mmu:54446+mmu:67087+mmu:81004	153	10	4	0.749
KEGG	PATHWAYS_IN_CANCER	5200	http://www.kegg.jp/kegg-bin/show_pathway?mmu05200+mmu:12402+mmu:12448+mmu:12540+mmu:14169+mmu:14369+mmu:16000+mmu:16842+mmu:17295+mmu:18590+mmu:18595+mmu:19730+mmu:22027+mmu:22339+mmu:22408+mmu:226519+mmu:26395+mmu:26417	323	17	3	0.726
KEGG	VEGF_SIGNALIN	4370	http://www.kegg.jp/kegg-bin/show_pathway?mmu04370+mmu:12540+	76	5	4	0.639

	G_PATHWAY		mmu:22339+mmu:26395+mmu:26417+mmu:54446				
KEGG	ERBB_SIGNALING_PATHWAY	4012	http://www.kegg.jp/kegg-bin/show_pathway?mmu04012+mmu:12325+mmu:12402+mmu:17974+mmu:18481+mmu:26395+mmu:26417	87	6	4	0.601
KEGG	MAPK_SIGNALING_PATHWAY	4010	http://www.kegg.jp/kegg-bin/show_pathway?mmu04010+mmu:12064+mmu:12290+mmu:12297+mmu:12540+mmu:14169+mmu:15511+mmu:16478+mmu:18590+mmu:18595+mmu:26395+mmu:26404+mmu:26409+mmu:26417	271	13	4	0.587
KEGG	SALIVARY_SECRETION	4970	http://www.kegg.jp/kegg-bin/show_pathway?mmu04970+mmu:12313+mmu:12314+mmu:22318+mmu:64177	77	4	3	0.585
KEGG	GLYCOLYSIS_GLUCONEOGENESIS	10	http://www.kegg.jp/kegg-bin/show_pathway?mmu00010+mmu:11674+mmu:16828	60	2	2	0.554
KEGG	B_CELL_RECEPTOR_SIGNALING_PATHWAY	4662	http://www.kegg.jp/kegg-bin/show_pathway?mmu04662+mmu:26395+mmu:26417+mmu:54446	76	3	4	0.539

Supplemental Table S3D. TLR4.

CATEGORY	TERM	TERM_ID	URL	TOTAL_GENES_OF_THE_TERM	UNION_TARGETS_IN_THE_TERM	MIRS_IN_THE_TERM	SCORE
KEGG	ADHERENS_JUNCTION	4520	http://www.kegg.jp/kegg-bin/show_pathway?mmu04520+mmu:11461+mmu:12388+mmu:13000+mmu:16842+mmu:17127+mmu:17295+mmu:21813+mmu:21872+mmu:22330+mmu:22612+mmu:26409+mmu:83767	74	12	4	2.242

KEGG	ARGININE_AND_PROLINE_METABOLISM	330	http://www.kegg.jp/kegg-bin/show_pathway?mmu00330+mmu:18451	53	2	2	1.931
KEGG	GLUTATHIONE_METABOLISM	480	http://www.kegg.jp/kegg-bin/show_pathway?mmu00480+mmu:18263+mmu:67305	54	2	2	1.864
KEGG	PATHWAYS_IN_CANCER	5200	http://www.kegg.jp/kegg-bin/show_pathway?mmu05200+mmu:112406+mmu:12395+mmu:12443+mmu:12448+mmu:12675+mmu:14369+mmu:16842+mmu:17127+mmu:17295+mmu:17311+mmu:18595+mmu:18710+mmu:19211+mmu:19730+mmu:21813+mmu:22027+mmu:22408+mmu:226519+mmu:26395+mmu:56458+mmu:58231	323	21	4	1.808
KEGG	WNT_SIGNALING_PATHWAY	4310	http://www.kegg.jp/kegg-bin/show_pathway?mmu04310+mmu:12325+mmu:12443+mmu:13000+mmu:14369+mmu:16842+mmu:17127+mmu:208846+mmu:22408+mmu:26409+mmu:26932+mmu:54446+mmu:67087+mmu:75826+mmu:81004	153	14	5	1.802
KEGG	AXON_GUIDANCE	4360	http://www.kegg.jp/kegg-bin/show_pathway?mmu04360+mmu:12632+mmu:12934+mmu:17295+mmu:18481+mmu:20315+mmu:20353+mmu:20355+mmu:20358+mmu:224105+mmu:268902+mmu:54446	131	11	5	1.669
KEGG	REGULATION_OF_ACTIN_CYTOSKELETON	4810	http://www.kegg.jp/kegg-bin/show_pathway?mmu04810+mmu:108083+mmu:11461+mmu:12632+mmu:17886+mmu:18481+mmu:18595+mmu:18710+mmu:19046+mmu:216963+mmu:21844+mmu:22330+mmu:224105+mmu:26395+mmu:66922+mmu:83767	215	15	5	1.504
KEGG	PROSTATE_CANCER	5215	http://www.kegg.jp/kegg-bin/show_pathway?mmu05215+mmu:12443+mmu:12448+mmu:12675+mmu:16842+mmu:18595+mmu:18710+mmu:19211+mmu:22027+mmu:26395+mmu:56458	89	10	3	1.363

KEGG	FOCAL_ADHESION	4510	http://www.kegg.jp/kegg-bin/show_pathway?mmu04510+mmu:11461+mmu:12443+mmu:17295+mmu:18481+mmu:18595+mmu:18710+mmu:19046+mmu:19211+mmu:22330+mmu:224105+mmu:226519+mmu:26395	197	12	4	1.265
KEGG	MAPK_SIGNALING_PATHWAY	4010	http://www.kegg.jp/kegg-bin/show_pathway?mmu04010+mmu:12064+mmu:12290+mmu:12294+mmu:12297+mmu:12675+mmu:18595+mmu:21813+mmu:224105+mmu:26395+mmu:26401+mmu:26404+mmu:26406+mmu:26409+mmu:58231+mmu:66922+mmu:67603	271	16	5	1.22
KEGG	ENDOCYTOSIS	4144	http://www.kegg.jp/kegg-bin/show_pathway?mmu04144+mmu:103967+mmu:13132+mmu:13858+mmu:16396+mmu:17127+mmu:17295+mmu:18595+mmu:216963+mmu:21813+mmu:72685+mmu:78618+mmu:98366	219	12	5	1.197
KEGG	COLORECTAL_CANCER	5210	http://www.kegg.jp/kegg-bin/show_pathway?mmu05210+mmu:12443+mmu:16842+mmu:17127+mmu:18710+mmu:19730+mmu:21813+mmu:26395	65	7	3	1.176
KEGG	T_CELL_RECEPTOR_SIGNALING_PATHWAY	4660	http://www.kegg.jp/kegg-bin/show_pathway?mmu04660+mmu:12675+mmu:18481+mmu:18710+mmu:224105+mmu:26395+mmu:26409+mmu:54446	109	7	4	1.071
KEGG	PANCREATIC_CANCER	5212	http://www.kegg.jp/kegg-bin/show_pathway?mmu05212+mmu:12443+mmu:12675+mmu:17127+mmu:18710+mmu:19730+mmu:21813+mmu:26395	70	7	3	1.051
KEGG	TIGHT_JUNCTION	4530	http://www.kegg.jp/kegg-bin/show_pathway?mmu04530+mmu:11461+mmu:13000+mmu:17879+mmu:17884+mmu:17886+mmu:18260+mmu:19211+mmu:21872+mmu:22612+mmu:66922	135	10	4	1.019
KEGG	RENAL_CELL_CARCINOMA	5211	http://www.kegg.jp/kegg-bin/show_pathway?mmu05211+mmu:112406+mmu:17295+mmu:18481+mmu:18710+mmu:224105+mmu:26395	71	6	3	1.017

KEGG	MELANOMA	5218	http://www.kegg.jp/kegg-bin/show_pathway?mmu05218+mmu:12443+mmu:17295+mmu:18595+mmu:18710+mmu:19211+mmu:26395	71	6	2	0.997
KEGG	BACTERIAL_INVASION_OF_EPITHELIAL_CELLS	5100	http://www.kegg.jp/kegg-bin/show_pathway?mmu05100+mmu:103967+mmu:11461+mmu:17295+mmu:18710+mmu:22330+mmu:83767	71	6	3	0.969
KEGG	CELL_ADHESION_MOLECULES_(CAMS)	4514	http://www.kegg.jp/kegg-bin/show_pathway?mmu04514+mmu:18260+mmu:20969+mmu:21367+mmu:320840+mmu:53321+mmu:54725+mmu:66797	149	7	4	0.946
KEGG	FC_GAMMA_R-MEDIATED_PHAGOCYTOSIS	4666	http://www.kegg.jp/kegg-bin/show_pathway?mmu04666+mmu:103967+mmu:12632+mmu:17118+mmu:17357+mmu:18710+mmu:26395+mmu:83767	90	7	3	0.92
KEGG	ACUTE_MYELOID_LEUKEMIA	5221	http://www.kegg.jp/kegg-bin/show_pathway?mmu05221+mmu:12395+mmu:12443+mmu:12675+mmu:16842+mmu:18710+mmu:26395	57	6	2	0.87
KEGG	P53_SIGNALING_PATHWAY	4115	http://www.kegg.jp/kegg-bin/show_pathway?mmu04115+mmu:12443+mmu:12448+mmu:12450+mmu:13663+mmu:18787+mmu:19211	69	6	2	0.863
KEGG	CHRONIC_MYELOID_LEUKEMIA	5220	http://www.kegg.jp/kegg-bin/show_pathway?mmu05220+mmu:12443+mmu:12675+mmu:17127+mmu:18710+mmu:21813+mmu:26395	73	6	3	0.848
KEGG	NOD-LIKE_RECEPTOR_SIGNALING_PATHWAY	4621	http://www.kegg.jp/kegg-bin/show_pathway?mmu04621+mmu:12675+mmu:21929+mmu:22027+mmu:26409+mmu:59079+mmu:66724	62	6	2	0.8
KEGG	GLIOMA	5214	http://www.kegg.jp/kegg-bin/show_pathway?mmu05214+mmu:12325+mmu:12443+mmu:18595+mmu:18710+mmu:19211+mmu:26395	65	6	3	0.794

KEGG	SMALL_CELL_LUNG_CANCER	5222	http://www.kegg.jp/kegg-bin/show_pathway?mmu05222+mmu:12443+mmu:12448+mmu:12675+mmu:18710+mmu:19211+mmu:226519	85	6	3	0.76
KEGG	MELANOGENESIS	4916	http://www.kegg.jp/kegg-bin/show_pathway?mmu04916+mmu:12325+mmu:14369+mmu:16842+mmu:17311+mmu:22408+mmu:26395	100	6	4	0.753
KEGG	GAP_JUNCTION	4540	http://www.kegg.jp/kegg-bin/show_pathway?mmu04540+mmu:14609+mmu:15560+mmu:18595+mmu:21872+mmu:26395	88	5	2	0.75
KEGG	ENDOMETRIAL_CANCER	5213	http://www.kegg.jp/kegg-bin/show_pathway?mmu05213+mmu:12443+mmu:16842+mmu:18710+mmu:19211+mmu:26395	52	5	2	0.74
KEGG	LEUKOCYTE_TRANSENDOTHELIAL_MIGRATION	4670	http://www.kegg.jp/kegg-bin/show_pathway?mmu04670+mmu:11461+mmu:12388+mmu:18260+mmu:18710+mmu:19229+mmu:20315+mmu:22330	120	7	4	0.728
KEGG	B_CELL_RECEPTOR_SIGNALING_PATHWAY	4662	http://www.kegg.jp/kegg-bin/show_pathway?mmu04662+mmu:12675+mmu:18710+mmu:26395+mmu:54446	76	4	4	0.678
KEGG	CELL_CYCLE	4110	http://www.kegg.jp/kegg-bin/show_pathway?mmu04110+mmu:12443+mmu:12448+mmu:13559+mmu:17127+mmu:19357+mmu:22628	125	6	4	0.644
KEGG	CYTOKINE-CYTOKINE_RECEPTOR_INTERACTION	4060	http://www.kegg.jp/kegg-bin/show_pathway?mmu04060+mmu:11479+mmu:11480+mmu:17295+mmu:17311+mmu:18383+mmu:18595+mmu:20315+mmu:21813	262	8	3	0.638
KEGG	ERBB_SIGNALING_PATHWAY	4012	http://www.kegg.jp/kegg-bin/show_pathway?mmu04012+mmu:12325+mmu:18481+mmu:18710+mmu:224105+mmu:26395	87	5	4	0.636
KEGG	TGF-BETA_SIGN	4350	http://www.kegg.jp/kegg-bin/show_pathway?mmu04350+mmu:11480+mmu:13559+mmu:17127+mmu:21813	85	4	3	0.635

	ALING_PATHWAY						
KEGG	PPAR_SIGNALING_PATHWAY	3320	http://www.kegg.jp/kegg-bin/show_pathway?mmu03320+mmu:14081+mmu:26569+mmu:50790	80	3	2	0.632
KEGG	NEUROTROPHIN_SIGNALING_PATHWAY	4722	http://www.kegg.jp/kegg-bin/show_pathway?mmu04722+mmu:12064+mmu:12325+mmu:18710+mmu:22628+mmu:26395+mmu:26401+mmu:26406	131	7	4	0.63
KEGG	NEUROACTIVE_LIGAND-RECEPTOR_INTERACTION	4080	http://www.kegg.jp/kegg-bin/show_pathway?mmu04080+mmu:107515+mmu:108073+mmu:11552+mmu:12311+mmu:12424+mmu:14401+mmu:15560+mmu:19219+mmu:19220	320	9	4	0.629
KEGG	GLYCOLYSIS_GLUCONEOGENESIS	10	http://www.kegg.jp/kegg-bin/show_pathway?mmu00010+mmu:11674+mmu:16828	60	2	2	0.62
KEGG	ARRHYTHMOGENIC_RIGHT_VENTRICULAR_CARDIOMYOPATHY_(ARVC)	5412	http://www.kegg.jp/kegg-bin/show_pathway?mmu05412+mmu:11461+mmu:12294+mmu:12297+mmu:14609+mmu:16842	74	5	3	0.605
KEGG	VEGF_SIGNALING_PATHWAY	4370	http://www.kegg.jp/kegg-bin/show_pathway?mmu04370+mmu:18710+mmu:26395+mmu:54446	76	3	4	0.594
KEGG	CALCIUM_SIGNALING_PATHWAY	4020	http://www.kegg.jp/kegg-bin/show_pathway?mmu04020+mmu:12290+mmu:12325+mmu:15560+mmu:18595+mmu:19220+mmu:19229	178	6	4	0.588
KEGG	CHAGAS_DISEASE	5142	http://www.kegg.jp/kegg-bin/show_pathway?mmu05142+mmu:12675+mmu:17127+mmu:18710+mmu:18787+mmu:21813	102	5	3	0.582
KEGG	OOCYTE_MEIOSIS	4114	http://www.kegg.jp/kegg-bin/show_pathway?mmu04114+mmu:12325+mmu	112	6	3	0.578

			:12448+mmu:19046+mmu:22628+mmu:26395+mmu:26932				
KEGG	PHOSPHATIDYLINOSITOL_SIGNALING_SYSTEM	4070	http://www.kegg.jp/kegg-bin/show_pathway?mmu04070+mmu:104418+mmu:108083+mmu:18710+mmu:19211+mmu:212111	78	5	2	0.557
KEGG	ADIPOCYTOKINE_SIGNALING_PATHWAY	4920	http://www.kegg.jp/kegg-bin/show_pathway?mmu04920+mmu:12675+mmu:14081+mmu:50790	68	3	3	0.541
KEGG	NON-SMALL_CELL_LUNG_CANCER	5223	http://www.kegg.jp/kegg-bin/show_pathway?mmu05223+mmu:12443+mmu:18710+mmu:26395+mmu:58231	54	4	2	0.515
KEGG	BASAL_CELL_CARCINOMA	5217	http://www.kegg.jp/kegg-bin/show_pathway?mmu05217+mmu:14369+mmu:16842+mmu:22408	55	3	2	0.506
